# Hard carbon from a sugar derivative for next-generation sodium-ion batteries†

**DOI:** 10.1039/d4mh01118j

**Published:** 2024-11-13

**Authors:** Enis Oğuzhan Eren, Evgeny Senokos, Zihan Song, Brinti Mondal, Audrey Perju, Tim Horner, Elif Begüm Yılmaz, Ernesto Scoppola, Pierre-Louis Taberna, Patrice Simon, Markus Antonietti, Paolo Giusto

**Affiliations:** a Department of Colloid Chemistry, Max Planck Institute of Colloids and Interfaces Potsdam 14476 Germany enis.eren@mpikg.mpg.de paolo.giusto@mpikg.mpg.de; b Department of Biomaterials, Max Planck Institute of Colloids and Interfaces Potsdam 14476 Germany; c Department of Engineering Science, University of Oxford Oxford OX1 3PJ UK; d Université Paul Sabatier, CIRIMAT UMR CNRS 5085 118 Route de Narbonne Toulouse 31062 France

## Abstract

Sodium-ion batteries have emerged as a promising secondary battery system due to the abundance of sodium resources. One of the boosters for accelerating the practical application of sodium-ion batteries is the innovation in anode materials. This study focuses on developing a high-performance hard carbon anode material derived from hydroxymethylfurfural, produced from carbohydrates, using a straightforward thermal condensation method. The process results in a unique pseudo-graphitic material with abundant microporosity. Electrochemical evaluations demonstrate excellent sodium storage performance by maintaining the plateau capacity even at higher current densities. This translates to a promising energy density when coupled with the cathode material. However, we also discuss the influence of electrolyte composition on the performance of the hydroxymethylfurfural-derived hard carbon, emphasizing the critical role of electrolyte optimization for the development of efficient and sustainable carbonaceous anode materials for next-generation sodium-based batteries.

## Abstract 2

We report on the synthesis of a hard carbon from hydroxymethylfurfural, a sugar derivative, highlighting its excellent potential as an anode material for next-generation sodium-ion batteries.

## Graphical abstract

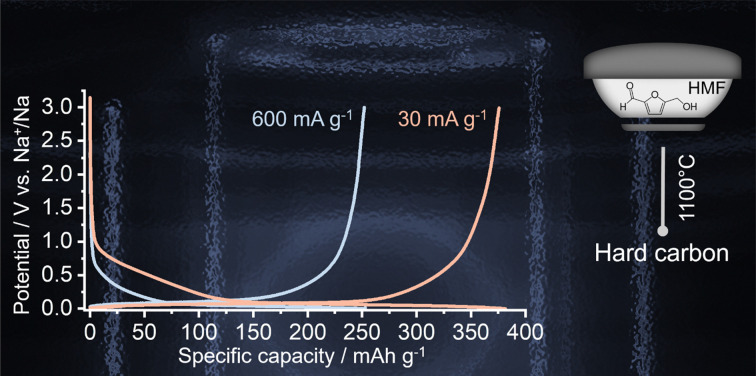

**New concepts**

In this manuscript, we introduce a high-performance hard carbon anode material designed for next-generation sodium-ion batteries. Our method involves the thermal condensation of a carbohydrate-derived precursor, specifically hydroxymethylfurfural. The resulting hard carbon material ranks among the best-performing hard carbon anodes, particularly when compared to similar carbons derived from hexose. Notably, it maintains excellent performance even at higher charging rates, making it ideal for fast-charging applications—an important advancement for sodium-ion batteries, which typically experience significant performance drops at such rates due to the slow kinetics of faradaic processes. Additionally, we critically examine the electrolyte and the adverse effects of a commonly used fluorinated electrolyte additive. Eventually, our findings highlight that adapting technology from other battery systems is not viable due to the unique sodium storage mechanisms in these carbons.

## 1. Introduction

For more than a decade, sodium-ion batteries (SIBs) appeared on the stage as a niche in energy research, eventually increasing the attention as alternative secondary battery systems to lithium–ion batteries (LIBs) due to their sustainability and affordability.^1^ Even though LIBs are the current benchmark for secondary batteries, their geopolitical and environmental implications suggest that they may not be the key player in the near future.^2^ Sodium is more abundant and homogeneously distributed in the crust and potentially available from several sources.^3^ However, SIBs are still mainly considered complementary to LIBs, with slightly lower performance metrics, and therefore, they have not yet been implemented on a large scale. Recent developments show that SIBs can potentially overturn this trend if appropriate battery pack-level energy densities are achieved.^4^ Overcoming the energy density barrier of 200 W h kg^−1^ is currently considered the target for entering the mobile application market. Indeed, the transition may start with small electric vehicles (*i.e.*, city cars that are not required to cover long distances) and eventually become a broadly applied substitute for LIBs.^4^ To achieve this, developing and implementing high-performance electrode materials is crucial.

The similarities between the working principles of LIBs and SIBs enable the implementation of analogous crystalline materials used in LIB chemistry on the cathode side. However, on the anode side, graphite does not provide sufficient performance due to the different physicochemical properties of sodium with respect to lithium, hindering the formation of a stable carbon–sodium intercalation compound.^5^ As a result, research on alternative high-performance anode materials has significantly intensified. In particular, inorganic anode materials such as Sn, metallic selenides, and hybrid materials have gained recognition as promising candidates for SIBs.^6^ Among the carbonaceous materials, hard carbons are considered one of the most promising solutions for anode materials in SIBs due, among others, to their turbostratic structure, providing a high volume of closed porosity.^7^ The exploitation of hard carbons as anode materials in SIBs has shown promising electrochemical energy storage performance, reaching specific capacity values of more than 300 mA h g^−1^ with a long plateau close to sodium's reduction potential.

The sodium storage mechanism in hard carbons has been deeply investigated, and the widely accepted description involves two main different stages.^8^ The first one involves a “sloping region”, which occurs at the beginning of the galvanostatic charge and discharge (GCD) profile and is attributed to a surface-controlled, capacitive-like storage mechanism arising from the surface adsorption of sodium ions. The second one, the diffusion-controlled “plateau region”, exhibits a nearly constant voltage profile close to 0 V (*vs.* Na^+^/Na) and accounts for the most significant contribution to the energy density of the sodium-storage mechanism.^7^ This stage involves the diffusion of sodium ions, particularly to the micropores. The plateau plays a critical role in achieving the goal of having high energy density in full-cell devices, as it secures a constant, high cell voltage when coupled with the cathode material.

Hydroxymethylfurfural (HMF) is an organic compound derived from the dehydration of sugars, primarily from fructose.^9^ It serves as a promising molecule in green chemistry due to its renewable origins and potential for conversion into valuable materials.^10^ While acknowledging the promising properties of HMF, the scalability and industrial feasibility of the synthesis process are critical factors for real-world implementation. One potential challenge in scaling up the production of HMF is the availability.^11^ However, recent advancements in biomass conversion technologies, such as improved catalytic processes^9^ and more efficient use of renewable feedstocks, are expected to reduce the cost and increase the availability of HMF production significantly.^11^ These innovations are making HMF a more economically viable precursor and renewable solution for large-scale applications. From an environmental perspective, HMF synthesis from biomass presents a more sustainable route compared to fossil-derived alternatives, with the potential for lower carbon emissions.^12^ Furthermore, the scalability of the thermal processes can be enhanced by integrating continuous-flow reactors,^11^ which can handle higher volumes with reduced energy inputs.

**Fig. 1:**
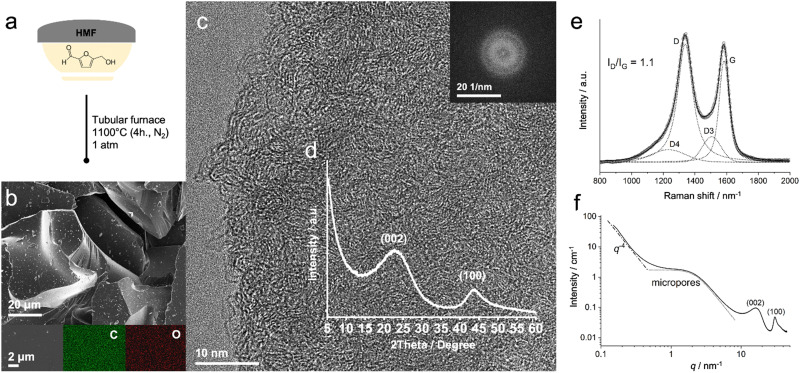
(a) Synthesis scheme of HMF-HC. (b) SEM image, along with oxygen and carbon EDX maps of the designated region. (c) HRTEM image reveals the short-range ordered structure of HMF-HC. (d) XRD pattern of HMF-HC. (e) Deconvoluted Raman spectra with intensity ratios of D- and G-band. (f) SAXS/WAXS profile of HMF-HC.

Herein, we report the synthesis of a hard carbon anode material, namely HMF-HC, *via* a one-step thermal condensation of HMF at 1100 °C, without the need for complex pore-forming agents (Fig. 1a). Hard carbons with similar electrochemical signatures have typically been synthesized at temperatures higher than 1100 °C (Table S1, ESI†).^13^ In contrast, our approach demonstrates that the unique structure can be achieved at a lower temperature, important for reducing the energy footprint in the synthesis of these carbons. Moreover, as a low melting point precursor, HMF offers advantages in the carbonization process by ensuring homogeneity in the resulting carbon structure and allowing better control over morphology and porosity. Its liquid nature also simplifies scaling for industrial applications, making the process more efficient and adaptable. The HMF-HC exhibits very good electrochemical performance both at low and high current densities, revealing a high plateau capacity in all cases. This extended plateau capacity contributes to achieving a high full-cell voltage when coupled with a cathode material, leading to a promising energy density. This material stands out in terms of synthesis simplicity and high performance, positioning it among the highest-performance anode materials for SIBs (Table S1, ESI†). Additionally, we systematically evaluate the electrochemical storage of this material with different electrolytes, which revealed superior performance in the presence of an ether-based electrolyte with respect to conventional carbonate-based electrolytes. Eventually, we deem that this work provides important insight for developing efficient and sustainable materials for the future of sodium-ion battery systems.

## 2. Results and discussion

### 2.1. Physicochemical features

The choice of HMF as a precursor is a well-informed decision based on its position in the condensation pathway of carbohydrates. The predicted outcomes from the C–H–O ternary phase diagram lead to the development of hard carbon with the desired properties. The HMF's C–H–O ratio (2 : 2 : 1) enables it to closely align with the CO removal line at 1100 °C, distinctly positioned within the carbon deposition region.^14^ The removal of CO and CO_2_, in turn, is expected to act as pore-forming during thermal condensation.

The thermal condensation of HMF is inspected with thermogravimetric analysis (TGA) coupled with mass spectrometry (MS) to get insight into the evolution of the carbon structure. At low temperatures (<200 °C), HMF undergoes condensation polymerization, leading to complex polymer structures.^15^ Increasing the temperature further causes the thermal condensation of the polymer accompanied by the release of C^+^, C_*x*_H_*y*_^+^, CO^+^, O_2_^+^, and CO_2_^+^ groups around 550 °C, eventually forming a hard carbon structure (Fig. S1, ESI†).

Scanning electron microscopy (SEM) images (Fig. S2, ESI†) reveal that the material condenses in the form of large particles characterized by distinct sharp edges and boundaries, typical for hard carbon structures (Fig. 1b and Fig. S2, ESI†).^16^ Energy-dispersive X-ray (EDX) spectroscopy confirms the homogeneous distribution of oxygen (2.5 ± 1.0 wt%) within the carbon (97.5 ± 1.0 wt%), in good agreement with the results obtained from elemental analysis and XPS (Table S2, ESI†). The latter also conveys the chemical configuration of the elements on the surface. Here, the carbon core level (Fig. S3a, ESI†) reveals the typical strong peak around 284.5 eV associated with the sp^2^-C, although with contributions from the sp^3^-C and C–H bonds.^17^ Additional peaks at 286.1 and 287.5 eV indicate the presence of C_*x*_O_*y*_ species, such as carbonyl and carboxylic functional groups.^18^

At the nanoscale, the structure is examined using high-resolution transmission electron microscopy (HRTEM). The obtained images reveal the short-range ordered nature of the hard carbon with localized pseudo-graphitic layers (Fig. 1c and Fig. S4, ESI†).^19^ The absence of long-range order is confirmed by a single diffuse ring in the fast Fourier transform (FFT) (Fig. 1c, inset) and X-ray diffraction (XRD) patterns. The diffraction pattern shows two broad peaks centered at around 23° and 44° that are attributed to the (002) and (100) diffractions of hard carbons, respectively (Fig. 1d and Fig. S5, ESI†), with an average interlayer distance of 0.39 nm.^20^ These broad peaks serve as confirmation of the short-range order present in the materials associated with the pseudo-graphitic carbons, which might indicate a less dense structure when compared to graphite. Furthermore, Raman spectroscopy shows the characteristic D and G bands with a peak intensity ratio (*I*_D_/*I*_G_) of 1.1 alongside a broad 2D region, typical of hard carbons (Fig. 1e and Fig. S6, ESI†).^21^

Porosity and surface area are critical parameters for the design of functional electrode materials. Gas physisorption with N_2_ and CO_2_ is carried out to evaluate the material's open porous structure. The N_2_ sorption measurement provides information on the mesoporous structure of the material and, according to both density-functional-theory (DFT) and the Brunauer–Emmett–Teller (BET) model, the available surface area is very low (*ca.* 13 to 17 m^2^ g^−1^), pointing to bigger particles with a low amount of mesopores (Fig. S7, ESI†). Although prior efforts have often aimed to achieve higher surface areas for anode materials, we believe that the low amount of mesopores positively affects the performance of SIBs.^22^ Indeed, we expect that this will help to reduce an excessive growth of the solid–electrolyte interphase (SEI) layer and boost initial coulombic efficiency (ICE), both associated with the irreversible consumption of electrolyte during the initial sodiation and being directly correlated to the electrolyte-available surface area.^22,23^ CO_2_ physisorption provides information on the distribution of open micropores and ultramicropores due to the smaller kinetic radius of CO_2_ at the measurement conditions.^24^ Analysis of the pore size distribution reveals that the material is mainly composed of micropores smaller than 0.8 nm. The volume of accessible ultramicropores (*d*_pore_ < 0.7 nm) is determined to be 0.12 cm^3^ g^−1^, contributing to a large surface area of 724 m^2^ g^−1^ (Fig. S8, ESI†). Overall, the presence of micropores is beneficial in facilitating the diffusion-controlled storage of sodium ions, while the negligible portion of larger pores enhances the first cycle efficiency by suppressing the SEI growth.^22,23^

The presence of structural features, such as closed porosity, sharp interfaces, and fractal structures, with nanometer or larger sizes are confirmed by means of small-angle X-ray scattering (SAXS), widely recognized as a robust technique for investigating topology in amorphous materials.^25^ The SAXS pattern reveals two main regions in the case of hard carbons: the first one, namely the Porod region, is associated with the scattering from sharp interfaces, such as macroscopic surfaces, corresponding to a particular slope close to *q*^−4^ at small-angles, followed by a region at intermediate *q* ranges (*i.e.*, 0.5 to 3 nm^−1^) often associated with meso- and micro-pores (Fig. 1f).^25,26^ Given that the material does not predominantly contain mesopores, as confirmed by gas physisorption analysis, the scattering profile indicates that HMF-HC possesses a substantial amount of bulk microporosity.

Microporosity significantly enhances the efficiency of sodium storage in hard carbons.^7^ The presence of micropores and ultramicropores allows for the selective sieving of solvated Na^+^ ions, which promotes desolvation and increases ionic clustering.^27^ This process elevates the Na^+^ concentration, facilitating rapid ion transport during sodiation and desodiation. Moreover, the exclusion of solvent molecules from the micropores creates additional storage sites for bare Na^+^, thereby enhancing the material's overall capacity.^27^ Consequently, tuning the pore structure emerges as a promising strategy to substantially improve sodium storage capacity while maintaining high rate capabilities. Specifically, a high amount of bulk microporosity is anticipated to enhance the diffusion-controlled sodium storage mechanism of HMF-HC.

### 2.2. Sodium storage performance of the material

The electrochemical sodium storage performance of HMF-HC is evaluated in half-cells with 1 M NaPF_6_ in a diglyme as an electrolyte solution. Based on the initial GCD cycle, the ICE of HMF-HC is obtained as 72%. A value of significant importance regarding dynamic reactions taking place in the initial cycle, which is mainly dominated by the SEI formation.^28^ This process is specifically designated to stabilize the potential window between the negative electrode and the electrolyte. In this case, the formation of SEI is related to the surface area that is available to the electrolyte.^16*a*^ The size of the Na^+^/diglyme solvation shell is reported to be bigger than 0.4 nm in diameter, and when including the PF_6_^−^ counter ions, it is expected to increase further.^29^ This means that when the pore entrance is smaller than the size of the solvated ions, the pore surface is not accessible to the electrolyte, reducing the irreversible processes, increasing the ICE, and avoiding battery failures due to drying.

**Fig. 2:**
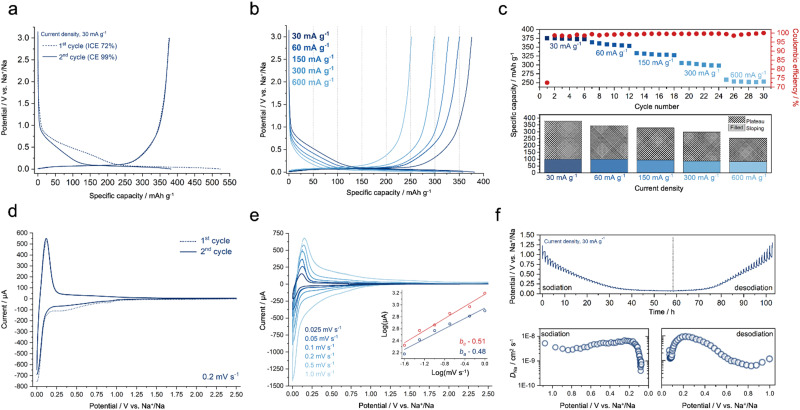
(a) Initial and second cycles, including coulombic efficiencies. (b) GCD profiles of HMF-HC at different current densities. (c) A detailed assessment of rate capability is provided, with a supplementary column chart depicting the distribution of “plateau” and “sloping” capacities at associated current densities. (d) Initial and second cycles with CV. (e) CV at different scan rates, with an inset displaying calculated *b*-values from both anodic and cathodic peaks. (f) GITT measurement, with a supplementary panel presenting sodium-ion diffusion coefficients in relation to sodiation and desodiation potentials.

Subsequent to the formation of an SEI layer, HMF-HC exhibits a reversible capacity of 376 mA h g^−1^ at a current density of 30 mA g^−1^ (Fig. 2a), in the same range as the theoretical capacity of graphite in LIBs (372 mA h g^−1^). Such a high reversible capacity, combined with a plateau capacity of almost 280 mA h g^−1^ (*ca.* 75% of total capacity), is achieved without the use of complex pore-forming templates, surface engineering methods, or post-synthesis processes, which points to the advantages of HMF-HC. In the following cycles, HMF-HC retains its initial reversible capacity, indicating also the absence of parasitic processes.

The HMF-HC is cycled at different current densities ranging from 30 mA g^−1^ to 600 mA g^−1^ (Fig. 2b) to convey how the electrochemical performances are affected by the charging rate. The material maintains a prolonged plateau capacity even at higher current densities, *e.g.*, beyond 300 mA g^−1^. HMF-HC displays a reversible capacity of 300 mA h g^−1^ at 300 mA g^−1^, retaining most of its plateau capacity at this rate, too. At 600 mA g^−1^, it still maintains a noticeable plateau, larger than 60% of the total capacity (Fig. 2c and Table S3, ESI†). These results are indeed impressive, especially if one considers that the hard carbons generally experience significant capacity decay at such high current densities due to the time-dependent nature of diffusion-controlled sodium storage.^30^

The sodium storage mechanism is investigated using the galvanostatic intermittent titration test (GITT) and cyclic voltammetry (CV). The initial CV curves in Fig. 2d indicate the presence of two major peaks at around 0.3 and 0.8 V (*vs.* Na^+^/Na), which are related to SEI formation during sodiation.^31^ Notably, these peaks are no longer present in the second cycle, suggesting the stability of the SEI and the absence of additional parasitic reactions. The temporal dynamics of sodium-ion concentration at the interface between the electrode and electrolyte, primarily manifest as time-dependent processes, which are therefore affected by the scan rate.^32^ The relationship between the logarithm of the peak anodic/cathodic current (*i*) and the logarithm of the scan rate (*v*) in CV plots, provides valuable information on the sodium storage mechanism. In particular, the slope denoted as the “*b*-value” in the log(*i*) *vs.* log(*v*) plots reveals the nature of the electrochemical process. A *b*-value close to 1 suggests a more surface-controlled mechanism, similar to electrical double-layer charge storage systems, while a *b*-value close to 0.5 indicates a more faradaic diffusion-controlled storage process.^32^ The calculated *b*-values from both anodic (0.48) and cathodic peaks (0.51) show that the diffusion-controlled mechanism is dominant in the overall sodium storage (Fig. 2e). Furthermore, from GITT measurements, it is possible to determine the sodium-ion diffusion coefficients within the material using the modified Fick's law of diffusion (Note S1, ESI†). This reveals that the sodium-ion diffusion coefficients of HMF-HC are consistent during the sodiation and desodiation processes. These diffusion coefficients are found to be in the order of 10^−8^ and 10^−9^ cm^2^ s^−1^ (Fig. 2f), which is in good agreement with previous reports on hard carbons.^33^ A significant decrease in the diffusion coefficients below 0.1 V (*vs.* Na^+^/Na) is also observed, attributed to the dominance of diffusion-controlled processes in the plateau region.

**Fig. 3:**
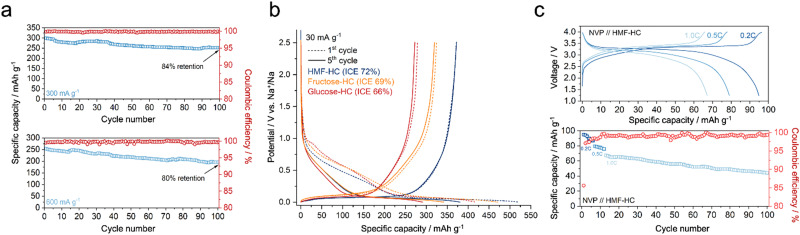
(a) Stability test at 300 and 600 mA g^−1^. (b) Comparative analysis of HMF-HC, glucose-HC, and fructose-HC. (c) Performance of the NVP//HMF-HC full-cell (working electrode: NVP) at various C-rates (based on the total battery capacity). The panel below demonstrates the cyclic stability of the full-cell at 1.0C.

It is worth recalling that the hard carbons in SIBs face inherent limitations in cyclic stability at higher current densities when compared to graphite in LIBs.^34^ The storage mechanism for hard carbons in SIBs primarily depends on aggressive ion insertion and pore-filling processes, which is detrimental to the structure and results in significant capacity decay in a relatively short time.^35^ The cycling stability of HMF-HC is evaluated at 300 mA g^−1^ and 600 mA g^−1^ current density, showing capacity retentions of 84% and 80% after 100 cycles, respectively (Fig. 3a). Several strategies have been explored to improve the cyclic stability of hard carbons, such as expanding the graphitic layers, introducing heteroatoms (*i.e.*, sulfur) into the carbon structure, or using more complex electrolytes.^36^ These strategies aim to mitigate the capacity fading issues and enhance the long-term stability of hard carbons in SIBs. However, for a high energy density full-cell, achieving a high cell voltage is essential. This is enabled by achieving an extended plateau region on the anode side.

As noted, HMF is a dehydration product of hexoses. Hence, we also compare HMF-HC to HCs derived from glucose and its isomer, fructose (Fig. 3b). While HCs derived from glucose and fructose have previously shown promising results in SIBs,^37^ we point out that their performances under the same conditions are notably inferior when compared to HMF-HC. The initial reversible capacity of the glucose-HC is found to be approximately 275 mA h g^−1^, while for fructose-HC, it is around 310 mA h g^−1^. Furthermore, the ICE values of the glucose-HC and fructose-HC are also slightly lower. We attribute this to a lower mass loss throughout carbonization and the related better preservation of the structure, while about 60 wt% is removed from glucose and fructose as water, only 30 wt% is eliminated in the case of HMF. The as-obtained chunks of material are then free of visible cracks and show a smooth surface, all of which enable a more efficient SEI formation and protection of the inside of the grains for effective energy storage. This improvement can also be observed during the initial sodiation process, where the SEI formation of glucose-HC and fructose-HC begins at slightly higher potentials (*vs.* Na^+^/Na) than that of HMF-derived hard carbon (HMF-HC), as shown in Fig. 3b.

**Fig. 4:**
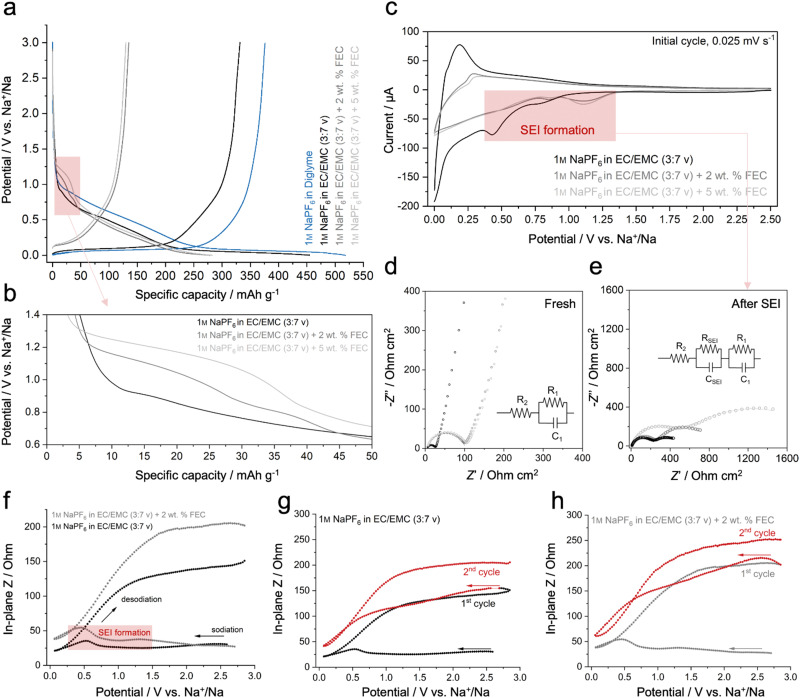
(a) Initial GCD cycle of HMF-HC in different electrolytes. (b) A detailed view highlights the alteration in electrolyte decomposition behavior with the inclusion of the FEC additive. (c) Initial cycle CV plots of HMF-HC in 1 M NaPF_6_ in EC/EMC with and without FEC additive. (d) Nyquist plots before SEI formation. (e) Nyquist plots after SEI formation. (f) *Operando* in-plane impedance analysis of HMF-HC in 1 M NaPF_6_ in EC/EMC with and without FEC additive, showing changes in the in-plane resistance in the initial cycle. In-plane resistivity changes during the initial and second cycles (g) without FEC additive and (h) with FEC additive.

The practical full-cell measurements are conducted by pairing HMF-HC with Na_3_V_2_(PO_4_)_3_ (NVP) cathode material. The characterizations regarding the NVP can be found in our previous study.^38^ The full-cell exhibits a long plateau, allowing for more than half of its capacity to be stored at a high voltage, near 3.4 V (Fig. 3c). This characteristic suggests a very high energy density of the full-cell device. It is worth noticing that in the full-cell experiments, we employ a carbonate-based electrolyte (1 M NaPF_6_ in EC/EMC (3 : 7 in vol%)) instead of the best-performing ether-based electrolyte due to operational constraints from the cathode side. Although the performance of HMF-HC with the carbonate-based electrolyte is slightly inferior (331 mA h g^−1^*vs.* 376 mA h g^−1^), it still shows a high capacity with a long plateau contribution (Fig. 4a). The full-cell demonstrates energy density values of 217 W h kg^−1^ at 0.2C (60 mA g^−1^ for HMF-HC), 176 W h kg^−1^ at 0.5C (150 mA g^−1^ for HMF-HC), and 146 W h kg^−1^ at 1.0C (300 mA g^−1^ for HMF-HC) (Table S5, ESI†), based on the active materials, and capacity retention of 67% at 1.0C after 100 charge/discharge cycles (Fig. 3c). The careful evaluation of the full-cell stability is crucial to convey its applicability, and we can expect to improve the stability further using more compatible electrolytes for the anode and the cathode.

### 2.3. Electrolyte compatibility and the effect of the fluoroethylene carbonate (FEC) additive

As previously mentioned, the performance of batteries is strongly dependent on the electrolytes and additives. For this reason, we have tested HMF-HC in different electrolyte environments in order to design strategies to improve the electrochemical performances further. In general, ether-based electrolytes have demonstrated notable improvements in sodium storage performance when compared to traditional carbonate-based electrolytes.^39^

Although the ionic conductivity (*ca.* 7 to 10 mS cm^−1^) and viscosity (*ca.* 0.7 to 1.1 cP) of diglyme and carbonates are relatively similar, diglyme demonstrates superior reductive stability.^40^ This is attributed to a higher lowest unoccupied molecular orbital (LUMO) level of ether-based solvents compared to carbonates,^40*b*^ which enhances the electrochemical stability window and makes diglyme less prone to decomposition. Another key advantage of diglyme is its ability to provide a lower coordination number for Na^+^ in the solvation shell. Typically, the solvation shell in diglyme is reported to have about two diglyme molecules per sodium ion (Na^+^[diglyme]_2_ complex), whereas carbonate-based electrolytes form larger solvation shells with 3 to 4 molecules.^41^ The higher dielectric constant of carbonate solvents (*i.e.*, 89.8 for EC)^40*b*^ also increases its polarity, creating a higher energy barrier for desolvation. This is critical for effective pore-filling mechanisms, as diffusion into closed pores and quasimetallic sodium clustering requires easy desolvation.

A comparative analysis of electrochemical impedance spectroscopy (EIS) reveals that the charge transfer resistivity of the cell with 1 M NaPF_6_ in diglyme is significantly lower than that of 1 M NaPF_6_ in EC/EMC (3 : 7 in vol%) (*R*_1_ in Fig. S10, ESI†) while providing similar electrode-associated resistance in both cases (*R*_2_ in Fig. S10, ESI†). This enhancement carries positive implications for the sodium-ion storage mechanism, potentially attributed to an improved desolvation process, which comes with a smooth transfer of sodium-ions through a thinner SEI layer.^42^ For example, the GCD curves highlight the different electrochemical performances of HMF-HC with 1 M NaPF_6_ in diglyme (Fig. 4a), achieving a reversible capacity of 376 mA h g^−1^ in the second cycle (Fig. S11, ESI†). On the other hand, the electrode with 1 M NaPF_6_ in EC/EMC (3 : 7 in v) displays a capacity of 331 mA h g^−1^ while maintaining similar ICEs in both cases. Moreover, the capacity increase is more pronounced within the plateau region, indicating that the ether-based electrolyte also improves the kinetics of the diffusion-controlled processes. The GCD results of HMF-HC in different electrolytes, obtained by systematically changing the salts, solvents, and additives, are summarized in Fig. S11 and Table S6 (ESI†).

The introduction of additives into the electrolyte solution has recently been acclaimed for improving the charge transfer and solvation/desolvation processes.^43^ Here, the well-known additive is fluoroethylene carbonate (FEC), which has shown promising results in increasing the performance of electrode materials and improving ICE, cyclic stability, and total capacity.^44^ However, a conflict arises concerning its compatibility with microporous anode materials.^45^ The formation of a thick and dense SEI layer when FEC is applied can hinder the potential diffusion channels to the bulk carbon, resulting in a capacity loss at the plateau and an overall decrease in performance.^45*c*,*d*,46^ Thus, despite the positive outcomes obtained with FEC in some applications, careful consideration and understanding of its effects are necessary for optimization.

Here, we employ our HMF-HC as a model to explore the effect of FEC additives on sodium storage in this class of materials. To the best of our knowledge, the use of FEC as an additive in ether-based electrolytes has not been reported, likely due to miscibility issues. In some cases, FEC can act as a potent co-solvent when combined with ether-based solvents, such as DME, in hybrid electrolytes for alkali metal batteries.^47^ However, we switched to a carbonate-based electrolyte (1 M NaPF_6_ in EC/EMC (3 : 7 in v)) to solely study the effect of FEC and to avoid potential incompatibilities between FEC and diglyme. Carbonate-based electrolytes, 1 M NaPF_6_ in EC/EMC (3 : 7 in vol%), with varying concentrations of FEC (0, 2, and 5 wt% FEC), are tested in a half-cell configuration. The GCD curves reveal a significant difference in the electrochemical signature in the presence and absence of the FEC additive at commonly reported concentrations (Fig. 4a). With the addition of FEC to the electrolyte solution, HMF-HC exhibits almost no plateau, resulting in a total capacity lower than 50% compared to the FEC-free electrolyte. Interestingly, variations in FEC amount do not significantly impact the outcomes, as both the addition of 2 and 5 wt% FEC lead to comparable results (Table S6, ESI†). To confirm that the performance decay is indeed attributed to the FEC additive, salt and solvent are changed while maintaining the FEC concentration (1 M NaPF_6_ in EC/DEC (1 : 1 in vol%) + 5 wt% FEC and 1 M NaClO_4_ in EC/PC (1 : 1 in vol%) + 5 wt% FEC) (Fig. S12, ESI†). In all cases, similar results are obtained. This confirms that the observed effect is an intrinsic behavior of FEC rather than a consequence of salt and solvent interactions (Table S6, ESI†).

The primary factor behind this phenomenon lies in the formation of a distinct SEI, which is present even in the initial sodiation cycle. In this context, our observations align with previous studies indicating that the rapid decomposition of FEC contributes to the formation of an inorganic, NaF-rich, and dense SEI layer due to the fluorine-rich electrolyte composition.^48^ The GCD curve illustrates that SEI-related electrolyte decomposition initiates at higher potentials when compared to the FEC-free electrolyte (Fig. 4b), a trend further emphasized in CV analysis (Fig. 4c). The FEC introduces two pronounced peaks around 0.8 and 1.2 V (*vs.* Na^+^/Na), contrasting with the FEC-free electrolyte, where the peaks occur at lower potentials, approximately 0.5 and 0.8 V (*vs.* Na^+^/Na). Also, a pair of redox peaks close to 0 V (*vs.* Na^+^/Na) appears suppressed with the addition of FEC.

Impedance spectroscopy is conducted at two state-of-charges to assess the charge transfer and SEI-related resistivity within the cell (Fig. 4d and e). Before SEI formation, the Nyquist plots of fresh cells exhibit a single semicircle associated with the electrode (*R*_2_) and charge transfer resistivity (*R*_1_). While *R*_2_ remains consistent, a noticeable difference in *R*_1_ is observed, indicating higher charge transfer resistance with the addition of FEC. Following the SEI formation (*ca.*, 0.5 V (*vs.* Na^+^/Na)), the Nyquist plots reveal double semicircles, a common occurrence linked to passivation-related resistivity.^49^ The distinctions among the electrolytes become clearer, significantly increasing the charge transfer and SEI-related resistivity (Fig. 4d and Table S7, ESI†) in the presence of the FEC additive. This further confirms that the performance decay may be directly related to the physicochemical properties of the SEI layer.

*Operando* in-plane impedance (*Z*) measurements are conducted to elucidate the effect of the FEC additive and monitor the resistivity changes during operation using a specialized setup, referred to as in-plane cell.^50^ In this configuration, the use of two potentiostats enables tracking the resistance/impedance changes (depending on the measurement frequency) of the electrode during sodiation and desodiation. Additionally, the in-plane geometry isolates the resistance/impedance measurement from the contributions of the counter and reference electrodes, the electrolyte, and the separator, thus enabling exclusive monitoring resistance/impedance of the working electrode.^50^ An initial inspection in this experimental setup is performed with CV analysis, revealing similar SEI-related peaks observed in a standard electrochemical cell (Fig. S13, ESI†).

Single frequency impedance measurements at 300 Hz are conducted to investigate the impedance changes during the SEI formation. At open circuit voltage (OCV), the in-plane impedances of the HMF-HC electrodes are found to be similar in electrolytes with and without FEC additive (Fig. S14, ESI†). During the initial sodiation step, the increase in in-plane impedance in the SEI formation region is more pronounced with the FEC-containing electrolyte (Fig. 4f, highlighted in the red transparent region). For example, a small peak around 1.2 V (*vs.* Na^+^/Na) observed in the CV (Fig. 4c), which is related to the decomposition of FEC, is also accompanied by increased impedance at the same potential (Fig. 4f). In-plane impedance measurements suggest that, during the initial sodiation cycle below 2 V (*vs.* Na^+^/Na), surface passivation of the electrode is significantly higher when compared to the FEC-free electrolyte. This further confirms that the fluorinated additive increases resistivity due to the formation of a distinct SEI layer. This increase in impedance is attributed to two simultaneous opposing effects: SEI formation, which increases impedance, and particle volume expansion, which decreases impedance by enhancing percolation within the plane. These competing effects limit the overall impedance change in the first cycle sodiation step. However, the increase in impedance related to the resistivity of the passivation layer is more pronounced with the FEC-containing electrolyte, indicating that SEI-related resistivity dominates over percolation.

In the second cycle, the SEI film is already formed and stable; therefore, no impedance increase is observed at the potentials associated with the SEI formation (Fig. 4g and h). The increase in impedance during desodiation is associated with the loss of contact between particles due to the volume change upon desodiation and the previously formed SEI layer. From the second cycle onwards, the irreversibility of impedance changes reduces notably, emphasizing the significant suppression of parasitic reactions associated with SEI formation.

An interesting feature observed during *operando* in-plane analysis is the slight decrease in impedance during sodiation, particularly when approaching the plateau region below 0.5 V (*vs.* Na^+^/Na) (Fig. 4f), where pore-filling occurs. This phenomenon has been previously observed,^51^ and although the exact cause remains unclear, we hypothesize that the introduced sodium ions enhance the overall conductivity of the carbon by increasing the carrier concentration. As the ions enter the carbon matrix, they improve electronic conductivity, reducing resistance. Additionally, as sodium ions occupy confined pore spaces, more efficient ionic pathways are created within the carbon structure, improving charge transfer.

Additional tests were carried out at different frequencies: 300 Hz, 3 kHz, and 0 Hz (direct current or DC) (Fig. S15, ESI†). The in-plane impedance at these frequencies changes similarly, showing the same pattern but with different magnitudes. DC measures mainly electronic impedance, and the consistent trend at higher frequencies suggests we observe variation in the electronic percolation.^50^ As frequency increases, the impedance decreases because the ionic component starts to influence the measurements. However, even at 3 kHz, the purely ionic variation cannot be measured, indicating that the sodiation/desodiation in the HMF-HC is primarily an electronically-driven process.^50^

**Fig. 5:**
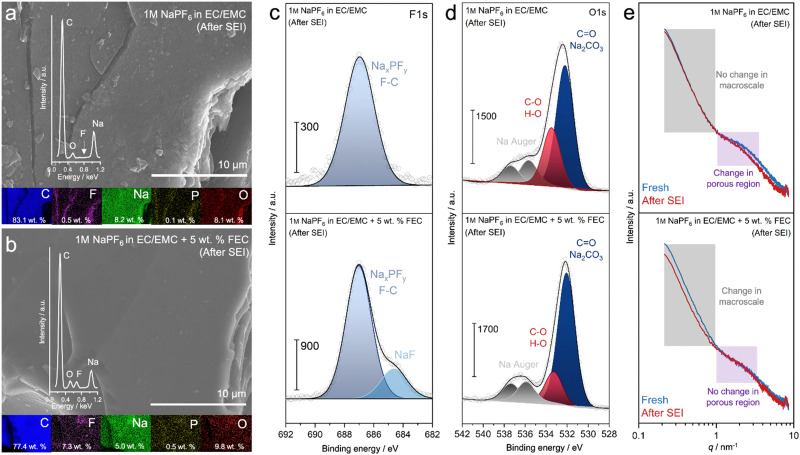
*Ex situ* SEM investigations of HMF-HC without (a) and with (b) FEC additive, show significant differences in the chemical compositions in relation to the SEI. Inset: EDX spectrum and mappings of C, F, Na, P, and O. *Ex situ* XPS analysis shows the variation in surface chemistry of HMF-HC after SEI formation in carbonate-based electrolytes with and without FEC additive: (c) F 1s core-level and (d) O 1s core-level, including Na Auger peaks.^53^ (e) *Ex situ* SAXS analysis reveals changes in the micro- and macro-scale structure of HMF-HC with and without the use of FEC additive.

The physicochemical features of SEI layers are characterized through *ex situ* SEM-EDX, XPS, and SAXS. SEM images indicate that the HMF-HC electrode cycled in the presence of FEC-containing electrolyte appears to be coated by a smooth film, whereas the FEC-free electrolyte presents some surface features that resemble one of the bare electrode materials (Fig. 5a). EDX spectra reveal a significantly lower fluorine content in the FEC-free electrolyte, pointing towards a minor decomposition of the electrolyte anion, which can be washed away from the electrode surface, leaving sodium and oxygen as the primary species. Conversely, FEC addition results in a homogeneous distribution of fluorine (*ca.* 7 wt%) on the surface, which shows that the fluorine here is an integral, structural part of the passivation layer (Fig. 5b). *Ex situ* XPS findings once more show that the electrode cycled in FEC-containing electrolyte exhibits a shoulder in the F 1s core level, indicative of NaF domination.^52^ Here, FEC can release F^−^ upon decomposition, where these fluoride ions can react with adsorbed Na^+^ at the surface of the hard carbon, eventually forming more NaF-dominated SEI. On the other side, strong contributions from NaF are absent in the FEC-free electrolyte, with signals primarily originating from Na_*x*_PF_*y*_ and F–C (Fig. 5c). At the C 1s core level, the carbon main peak exhibits a reduced signal in the presence of FEC, which can be attributed to a thicker SEI (Fig. S16, ESI†). Additionally, the carbonate features in the C 1s core level (*ca.* 290 eV) display a more pronounced peak with FEC, providing additional evidence of the presence of more inorganic substances in the SEI, such as Na_2_CO_3_ (Fig. S16 and Table S8, ESI†).^48^ This is further supported by the O 1s core level, where the peak related to metal–carbonates (*ca.* 532 eV) becomes much sharper with the presence of the FEC additive (Fig. 5d).^53^ NaF forms at relatively higher potentials and is known for its excellent mechanical properties, electrochemical stability, and ionic conductivity, making it robust and stable over long cycling periods.^54^ Its ability to extend the electrochemical window to very high potentials is particularly beneficial for cathode materials. In contrast, Na_2_CO_3_ is more flexible, better accommodating volume expansion, and forms at lower voltages, which can contribute to improved initial cycling stability. However, due to its lower stability and higher solubility in EC-based solvents,^55^ Na_2_CO_3_ may gradually dissolve during cycling, leading to increased electrolyte consumption and parasitic reactions. The intrinsic effects of these compounds, along with Na_2_O, which is part of the mosaic SEI concept, are difficult to deconvolute. The complex sodium storage mechanisms observed in hard carbons appear incompatible with SEI layers that are rich in such inorganic compounds.

The structural changes are also monitored with *ex situ* SAXS experiments, differentiating them from macro to micro levels (Fig. 5e and Fig. S17, ESI†). In the mid-*q* range, a wide scattering distribution arises from micro and mesopores, while smaller *q* values indicate macroscale changes, specifically interfacial changes. With FEC-free electrolyte, changes in the macroscale are not apparent as the Porod slope and intensity remain similar, suggesting a minimal effect on the interface. However, a slight intensity decrease in the mid-*q* region indicates that the SEI-related changes occur instead at the pore level, a phenomenon observed in a previous study.^56^ In contrast, adding FEC does not cause any alterations in the microporous region. Instead, a clear change in the Porod slope is observed, pointing to a change in the macroscale assemblies, which could arise from a thicker SEI formation. A change in the Porod slope also suggests a change in the electron density contrast and surface roughness between the carbon and a thick SEI layer. A porous or inhomogeneous interphase composed of organic and/or inorganic sodium compounds can introduce additional surface features and roughness, causing the Porod slope to become less steep. Furthermore, a thick SEI layer has a higher chance of creating a composite interface with varying density regions, altering the overall scattering profile at a low *q* range and thus changing the Porod slope. This emphasizes that the formation of a NaF-rich and dense SEI has a severe impact on the morphology and electrochemical behavior of the hard carbon anode.

As stated earlier, the sodium storage mechanism in hard carbons is typically dominated by adsorption in the slope region and pore-filling in the plateau region. In our previous study, *operando* small-angle X-ray scattering (SAXS) analysis showed that the micropore-filling mechanism occurs in the plateau region.^38^ In this study, incorporating FEC as an additive led to the formation of a dense SEI layer, which preserved bulk porosity but blocked potential diffusion channels to those micropores. As a result, diffusion of sodium ions is hindered, reducing the plateau capacity and shifting the mechanism to surface-controlled capacitive behavior. This suggests that the dense SEI layer mostly affects the diffusion-controlled pore-filling process.

We invite for a careful evaluation of FEC additives in carbonaceous anode materials. The intricate dynamics become apparent when the anode possesses a substantial population of closed pores. In such cases, the formation of a thick and dense FEC-derived interphase may potentially obstruct the closed pores, resulting in a notable decrease in performance. The technology adopted from different battery systems or materials should be carefully evaluated before investigating the sodium storage mechanisms, especially in hard carbons.

## 3. Conclusions

A comprehensive investigation of the physicochemical features and sodium storage performance of HMF-derived hard carbon provides valuable insight into its potential as a promising anode material for SIBs. The thermal condensation process of HMF results in the formation of a hard carbon characterized by a unique microstructure with short-range order and abundant microporosity. The absence of mesopores and the dominance of ultramicropores contribute to enhanced sodium storage performances. The electrochemical evaluation of HMF-HC demonstrates excellent sodium storage capabilities, with a reversible capacity of 376 mA h g^−1^ at 30 mA g^−1^ current density and large plateau capacity even at higher rates (*ca.*, >70% of total capacity). When coupled with the cathode material, the full-cell exhibits a long plateau at about 3.4 V, which translates to an energy density of 217 W h kg^−1^, making this material of potential interest even for large-scale applications. Additionally, the exploration of electrolyte compatibility highlights the importance of solvents and additives in accessing the full electrochemical performance of HMF-HC. The FEC-containing electrolyte adversely impacts the sodium storage performance of HMF-HC, leading to the formation of a dense and NaF-rich SEI. This increases the charge transfer resistance, resulting in a capacity decay, emphasizing the critical role of electrolytes in determining the limit of such microporous anode materials. Eventually, we aim to develop more efficient anode materials from more sustainable, renewable, circular, and available sources as one of the critical parameters to design the next generation of high-energy density SIBs.

## 4. Experimental

### 4.1 Synthesis

2 g of 5-(hydroxymethyl)furfural (HMF, Sigma-Aldrich, >99%) was introduced into an alumina crucible (5 cm in diameter and 3 cm in height). Subsequently, the crucible was placed in a tubular furnace. The temperature of the furnace was gradually increased at a rate of 10 °C per minute until it reached 1100 °C under a nitrogen flow rate of 50 sccm. The furnace was maintained at this temperature for 4 hours. The resulting carbon material, denoted as HMF-HC, demonstrated a measured yield of approximately 30%. The final powder was further ground using conventional mortar for electrode preparation. In order to establish a comparative analysis, hard carbons derived from d-(+)-glucose (Sigma-Aldrich, >99%) and d-(−)-fructose (Sigma-Aldrich, >99%) were also synthesized with the same recipe.

### 4.2 Electrode preparation

The electrodes were produced through a process that involves blending HMF-HC with conductive carbon black (Super-P, Alfa Aesar) and polyvinylidene difluoride (PVDF, Kynar HSV-900) as the binding agent in a weight ratio of 80 : 15 : 5. The PVDF was dissolved in *N*-methylpyrrolidone (NMP, Sigma-Aldrich). The mixture was applied onto an aluminum foil using an automatic film applicator (mtv messtechnik, Germany) and dried under vacuum at 80 °C overnight. The final mass loading of the active material was around 1.2 mg cm^−2^.

### 4.3 General electrochemical measurements

Electrochemical measurements were conducted with three-electrode Swagelok-type cells on a Biologic MPG-2 instrument (France). The Swagelok-type cells were assembled in an argon-filled glovebox (MBRAUN, Germany) with an H_2_O and O_2_ content of less than 0.1 ppm. The electrolytes used in this study were acquired from E-Lyte GmbH. Glass fiber (Whatman GF/C) was used as a separator, and a thin slice of sodium metal (99.5%, Sigma-Aldrich) as both counter and reference electrode. The galvanostatic charge–discharge curves of the half-cells were collected in the potential range 0–3.0 V (*vs.* Na^+^/Na), with half-cells resting for six hours before the measurements. Cyclic voltammetry (CV) measurements were carried out at scan rates of 0.025, 0.05, 0.1, 0.2, 0.5, and 1.0 mV s^−1^ in the potential range 0.01–2.5 V (*vs.* Na^+^/Na). Electrochemical impedance spectroscopy (EIS) was performed with an AC perturbation of 10 mV in the frequency range 0.1 Hz to 20 kHz. The galvanostatic intermittent titration technique (GITT) was used to calculate the sodium-ion diffusion coefficients, where current pulses (30 mA g^−1^) were applied for 1200 s, and relaxation potentials were measured for 3600 s. For the full-cell study, the anode-to-cathode active material ratio was calculated as 1 : 3, excluding the losses in the initial cycle, as the specific capacity of HMF-HC is approximately three times that of NVP. Full-cell data was collected using two-electrode Swagelok-type cells. The physicochemical and electrochemical characterization of the NVP cathode was conducted in our previous study.^38^

### 4.4 *Operando* in-plane impedance analysis

The principle of the in plane EIS measurements has been described elsewhere.^50^ In short, electrode composed of 80% HMF active material, 10% conductive carbon additive and 10% PTFE were deposited onto an electronically insulating substrate, and dried in vacuum oven. The dried electrodes were further assembled inside a glove box and four metallic contacts were put in contact with the electrode to connect the two potentiostats. The dried electrodes were further assembled inside a glove box. Sodium metal was used as the counter and reference electrode, and GF/C was used as a separator. We performed the electrochemical measurements using 1 M NaPF_6_ in EC/EMC (3 : 7 v) and 1 M NaPF_6_ in EC/EMC (3 : 7 v) + 2 wt% FEC. The cells were cycled at a scan rate of 0.025 mV s^−1^ with a Biologic (France) potentiostat, and *operando* measurements were recorded at 0 Hz (DC), 300 Hz, and 3 kHz with a Gamry (USA) potentiostat in floating mode.

### 4.5 Physicochemical characterizations

The crystallinity of the samples was determined by X-ray diffraction (XRD) using the Rigaku SmartLab (Japan, Cu Kα, 0.154 nm). SAXS/WAXS measurements of the HMF-HC were conducted on the μSpot beamline of BESSY-II (Helmholtz-Zentrum Berlin, HZB, Germany).^57^ Experiments were performed using a monochromatic X-ray beam at 18.0 keV and a beam size of approximately 30 μm width obtained by a sequence of pinholes. The scattered intensities were collected with a Dectris Eiger 9 M detector. The scattering *q*-range was calibrated against quartz. For *ex situ* SAXS measurements, Bruker Nanostar II (USA, Cu K_α_, 0.154 nm) was used, and the sample-detector distance was 283 mm. Silver behenate was used for calibration. X-ray photoelectron spectroscopy (XPS) spectra were measured with a Phoibos 150 analyzer (Specs) using an Al K_α_ X-ray source (*hυ* = 1486.0 eV). The high-resolution spectra were recorded at pass energies of 35 eV. Raman spectroscopy of the HMF-HC powder was obtained using the WITec Alpha 300R (Germany) confocal Raman microscope with a laser wavelength of 532 nm. Laser energy was calibrated using a Si calibration sample. A laser power of 10 mW was selected for the acquisitions with a 20-s integration time. Data refinement was conducted by subtracting the background using WITec Project Five 5.2 software. The D and G bands were fitted with the PseudoVoigt model. Thermogravimetric analysis coupled with mass spectrometry (TGA-MS) was performed using a NETZSCH TG-209 Libra (Germany) under a helium atmosphere at a heating rate of 2.5 K min^−1^. Elemental analysis was performed using an Elementar Vario EL III (Germany), employing two different modes: a combustive mode for carbon, hydrogen, nitrogen, and sulfur; and a non-combustive mode for oxygen. Physisorption measurements were performed on a Quantachrome Quadrasorb SI (Austria) at 273 K for CO_2_ and 77 K for N_2_, with samples degassed overnight before measurement. The non-local density functional theory (NLDFT) method was employed to evaluate the pore size distribution (PSD). Elemental analysis was conducted using the Elementar Vario EL III (Germany). Scanning electron microscopy (SEM) imaging was performed using the Zeiss LEO 1550-Gemini (Germany) system with acceleration voltages of 3, 5, and 10 kV, and the energy-dispersive X-ray (EDX) data were collected using an Oxford Instruments X-MAX (UK) 80 mm^2^ detector. High-resolution transmission electron microscopy (HRTEM) was conducted using a Jeol JEM F200 (Japan) microscope.

### 4.6 Electrode preparation for *ex situ* physicochemical measurements

To facilitate *ex situ* measurements, electrode samples were prepared separately from the electrochemical characterizations. In order to enhance clarity and insight, we excluded contributions from conductive carbon (Super-P) and the binder (PVDF). In this particular case, HMF-HC powder was directly cold-pressed onto an aluminum current collector, achieving a mass loading similar to that obtained in regular electrochemical characterizations. A specially sealed container was used during sample transport for *ex situ* SEM-EDX samples. Similarly, for *ex situ* SAXS measurements, cycled electrodes were sealed between two Kapton films within a Glovebox. Each measurement spot on the electrode was mapped through the transmission and marked to provide consistency in reproducibility. The Kapton background does not interfere with the small-angle profile of the electrodes; thus, it was subtracted successfully.

## Author contributions

EOE performed the characterizations, synthesis of the materials, and wrote the original manuscript. ES, ZS, TH, and EBY contributed to the formal analysis of the physicochemical and electrochemical characterizations. ES conducted synchrotron SAXS experiments and contributed to the formal analysis. BM and AP performed the *operando* in-plane impedance measurements and participated in the formal analysis. PLT and PS conceptualized the *operando* in-plane impedance measurements. MA supervised the study and discussion. PG was responsible for conceptualizing and supervising the work.

## Data availability

The data supporting this article have been included as part of the ESI.†

## Conflicts of interest

There are no conflicts to declare.

## Supplementary Material

MH-012-D4MH01118J-s001
