# Supplementary material for: Hard carbon from a sugar derivative for next-generation sodium-ion batteries
Source: Mater Horiz. 2024 Nov 13;12(3):886–98. doi: 10.1039/d4mh01118j (PMC11563197; doi:10.1039/d4mh01118j)
Supplement: MH-012-D4MH01118J-s001 [file MH-012-D4MH01118J-s001.pdf]

Supplementary Information (SI) for Materials Horizons.
This journal is © The Royal Society of Chemistry 2024

# Supplementary Information

## Hard carbon from a sugar derivative for next-generation sodium-ion batteries

*Enis Oğuzhan Eren,*[a] Evgeny Senokos,[a] Zihan Song,[a,c] Brinti Mondal,[d] Audrey Perju,[d] Tim Horner,[a] Elif Begüm Yılmaz,[a] Ernesto Scoppola,[b] Pierre-Louis Taberna,[d] Patrice Simon,[d] Markus Antonietti,[a] and Paolo Giusto*[a]*

[a] *Department of Colloid Chemistry, Max Planck Institute of Colloids and Interfaces, Potsdam 14476, Germany*

[b] *Department of Biomaterials, Max Planck Institute of Colloids and Interfaces, Potsdam 14476, Germany*

[c] *Department of Engineering Science, University of Oxford, Oxford OX1 3PJ, United Kingdom*

[d] *Université Paul Sabatier, CIRIMAT UMR CNRS 5085, 118 Route de Narbonne, 31062, Toulouse, France*

**E-Mail: paolo.giusto@mpikg.mpg.de, enis.eren@mpikg.mpg.de*

## Supplementary Notes

**Supplementary Note 1.** We utilized the initial approximation method suggested by Weppner and Huggins,[1] which is applicable to both spherical and planar geometries.[2] Nevertheless, it is crucial to emphasize that this method is suitable for hard carbons, yet it requires specific assumptions.[3]

i. Step time is significantly less than the effective diffusion time.

ii. Transient data must be large enough not to include ohmic and kinetic overpotential.

Based on the single pulse GITT curve, a linear correlation exists between the potential response and the square root of the step time (**Figure S9**). Consequently, the first-order approximation can be expressed in a simplified form as the following equation.[3a, 3b]

$$D_{Na} = \frac{4}{\pi\tau}\left(\frac{m_B V_M}{M_B S}\right)^2\left(\frac{\Delta E_s}{\Delta E_\tau}\right)^2 \quad (1)$$

Where $\tau$ is the pulse duration, $m_B$ and $M_B$ are the actual and molar mass of the active material, $V_M$ is the molar volume, and *S* is the surface area of the electrodes. $\Delta E_s$ (change of the steady-state voltage during a single-step GITT curve) and $\Delta E_\tau$ (change of cell voltage during a constant current pulse) can be extracted from the typical GITT curve of the material.[3d, 4] The sodium ion diffusion coefficients of the materials are summarized in the manuscript.

**Supplementary Note 2.** The energy density of HMF-HC//NVP is determined using the following equation, where *Q* is the capacity at a given rate, *m* is the mass of the total active materials, and *U* is the cell voltage (Eq. 2).

$$E = \int_0^Q \frac{U}{m} dQ \quad (2)$$

## Supplementary Figures

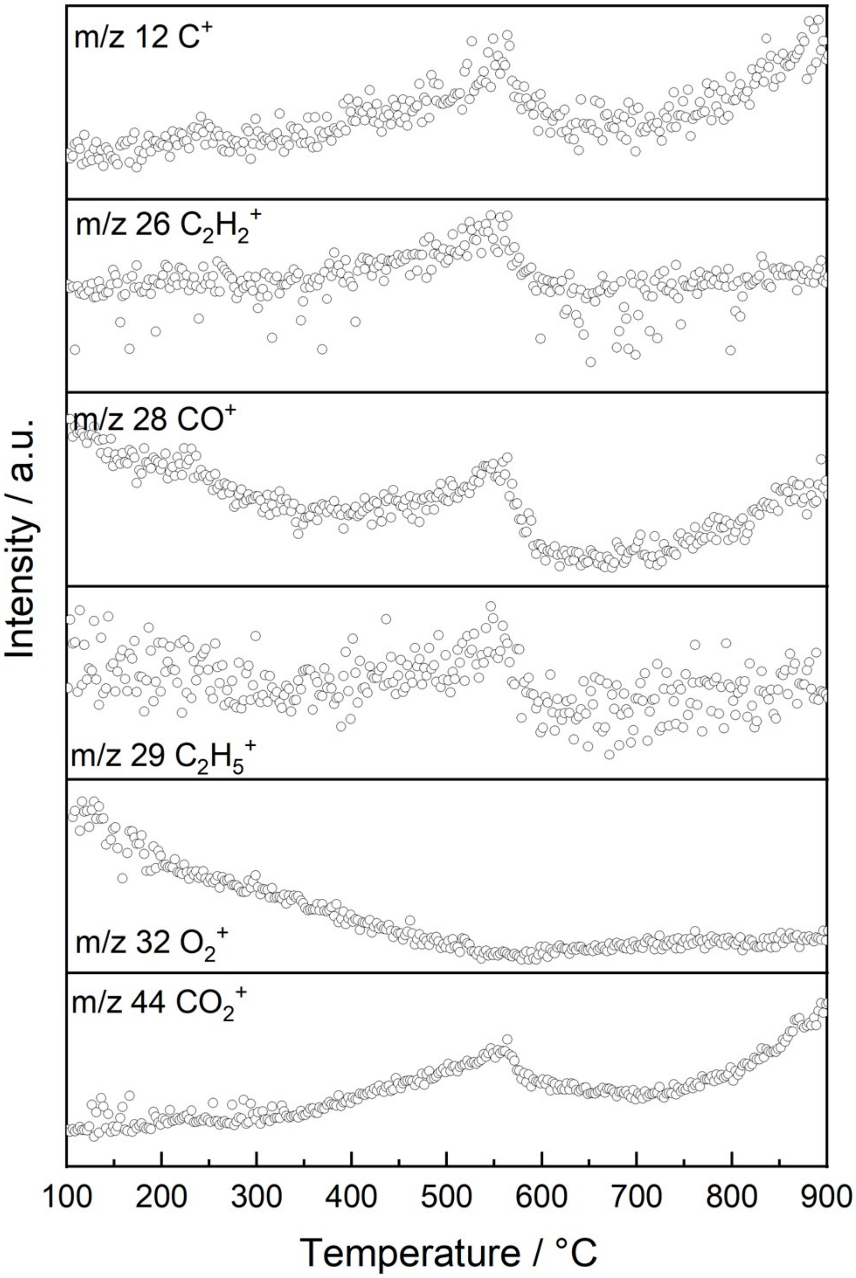


**Figure S1**. The m/z values significantly change during TGA-MS measurement under a helium atmosphere.

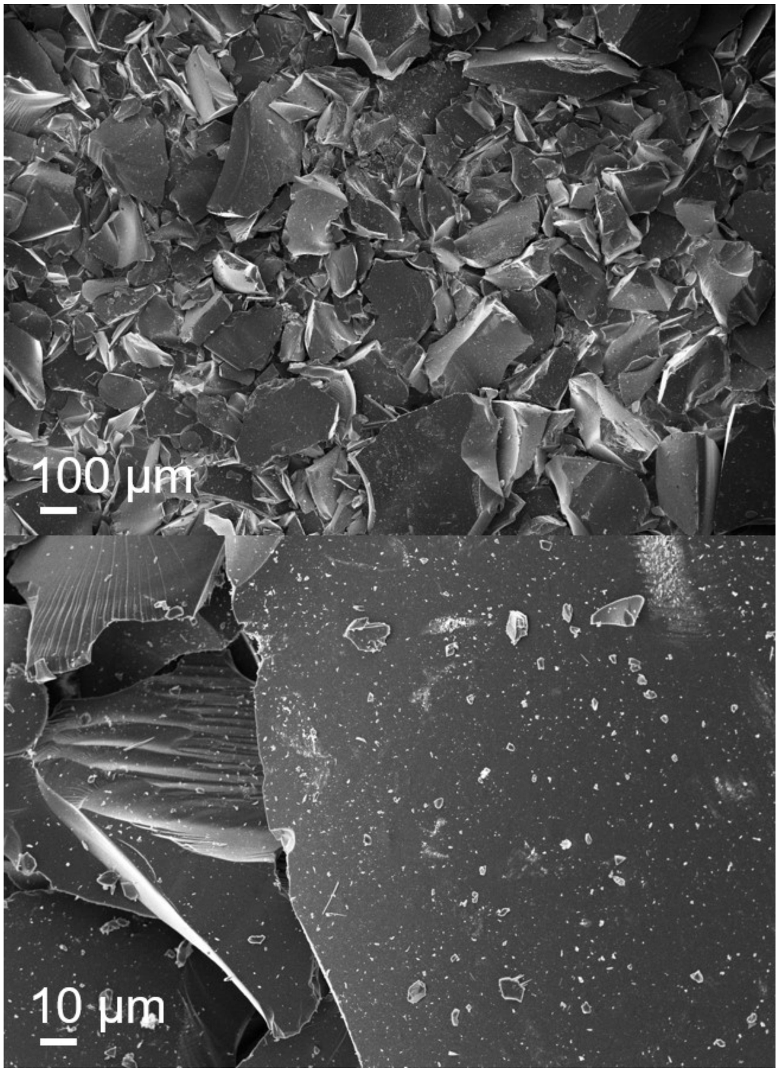


**Figure S2**. SEM images of the as-prepared HMF-HC powder at different magnifications.

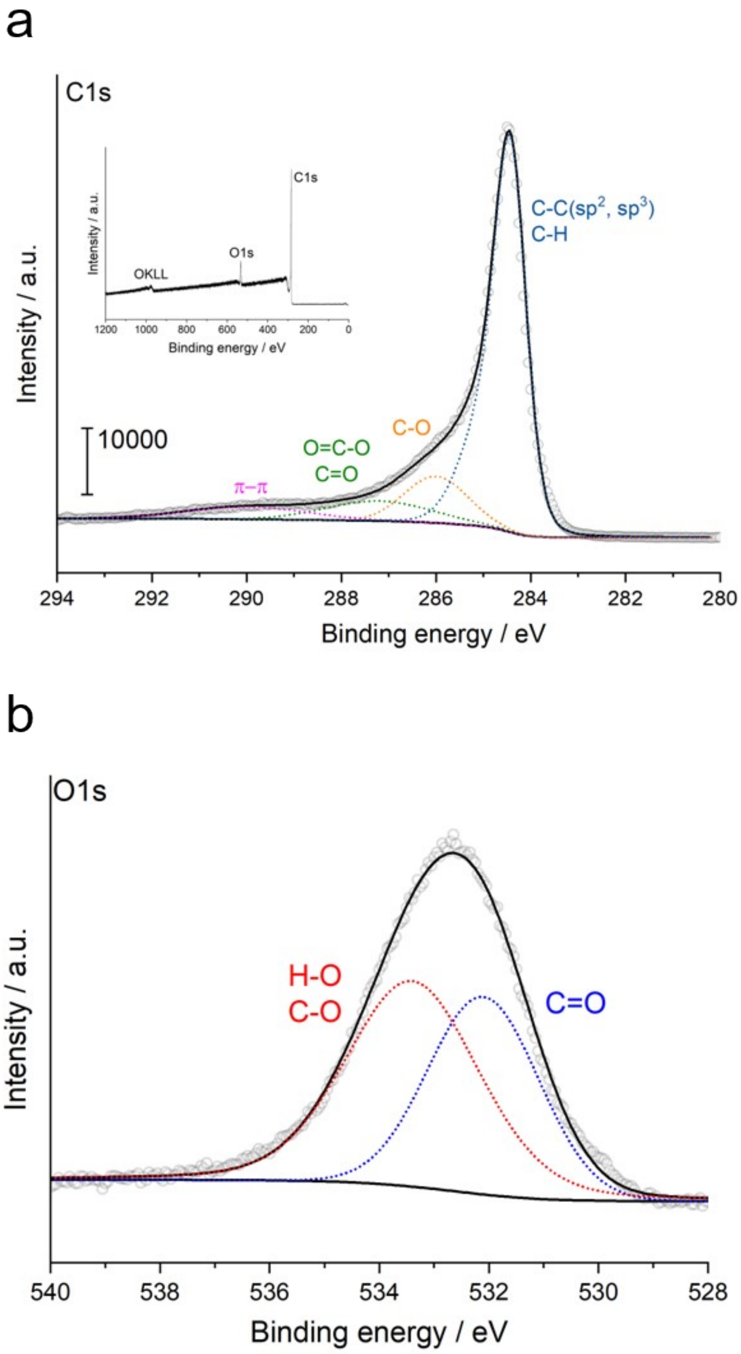


**Figure S3**. XPS spectra of HMF-HC. (a) Deconvoluted C1s core-level. Inset: Full survey. (b) O1s core-level.

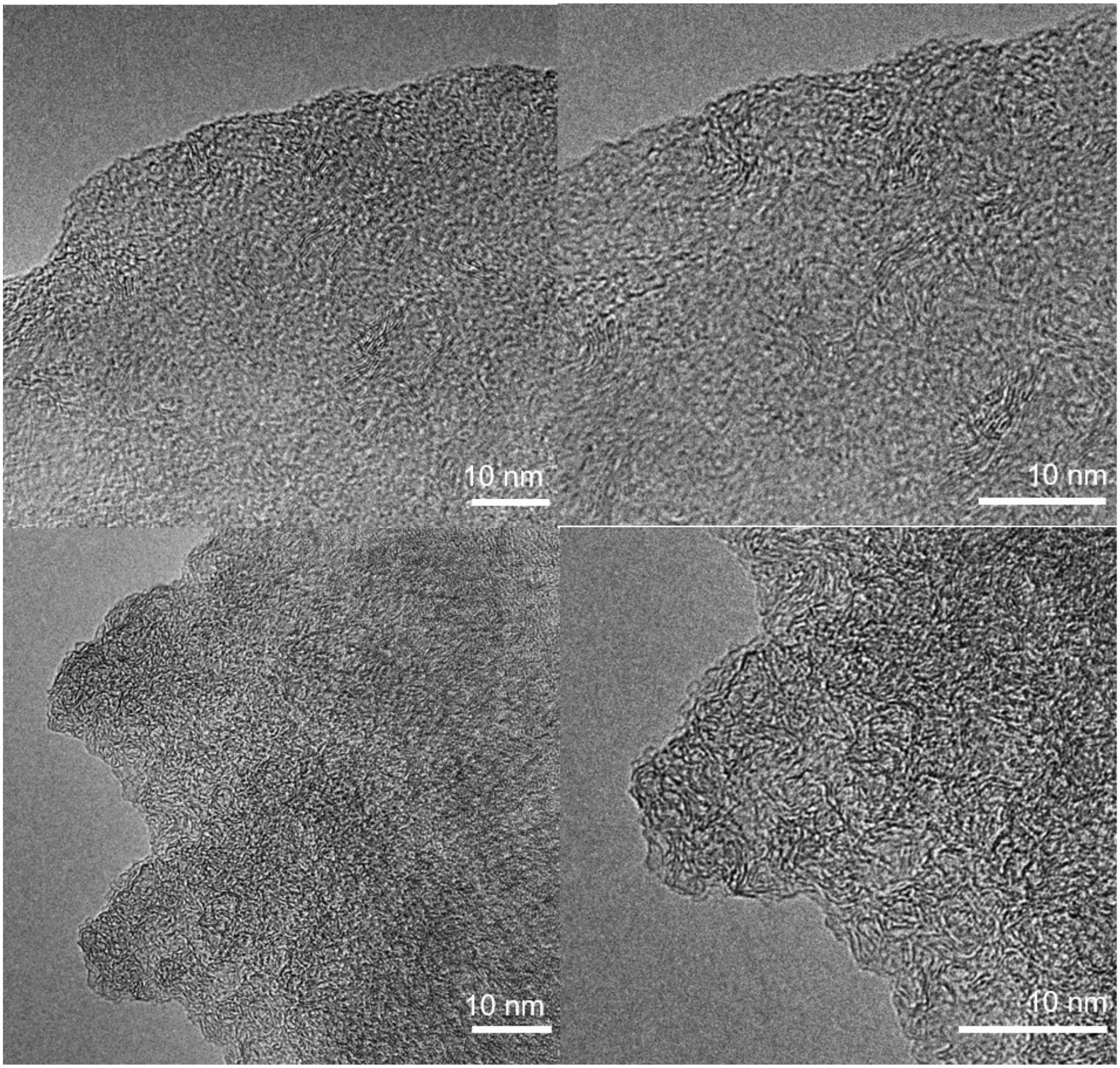


**Figure S4**. HRTEM images of the HMF-HC at different magnifications show the typical short-range order of the non-graphitic carbons at the nanoscale.

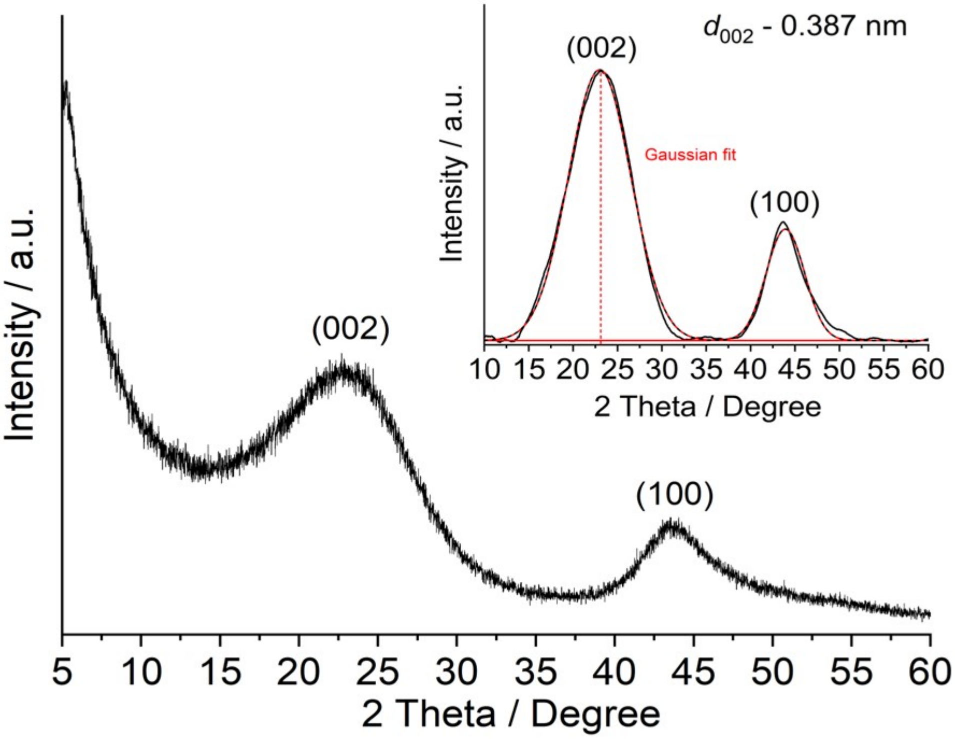


**Figure S5**. XRD pattern of the HMF-HC. Inset: Gaussian model is fitted for (002) and (100) to calculate interlayer spacing from Bragg's equation.

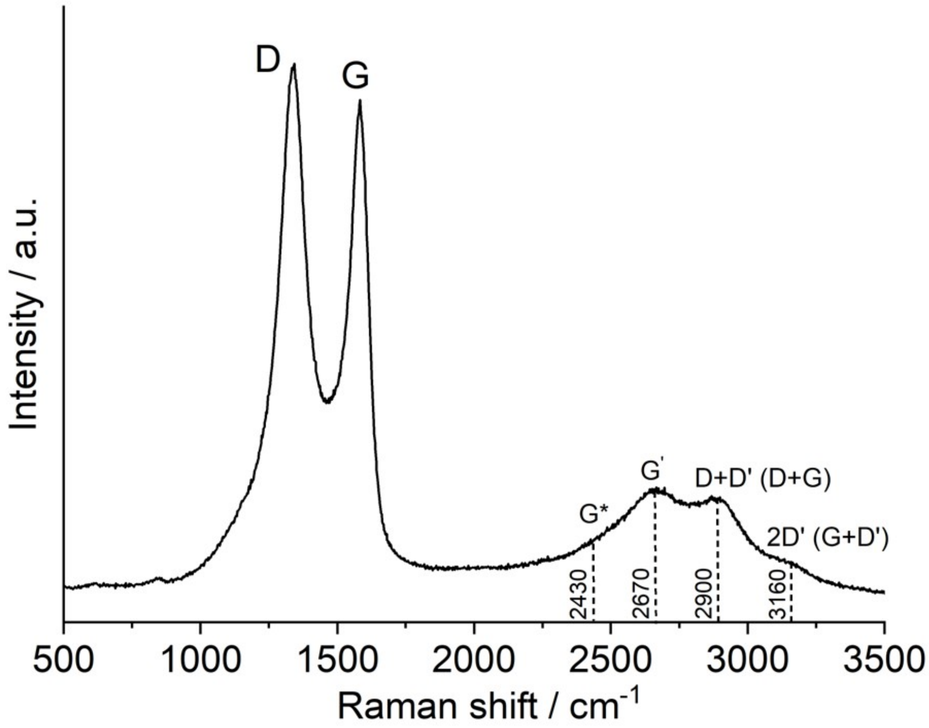


**Figure S6**. Raman spectra of the HMF-HC with a broad 2D region associated with the non-graphitic carbons.

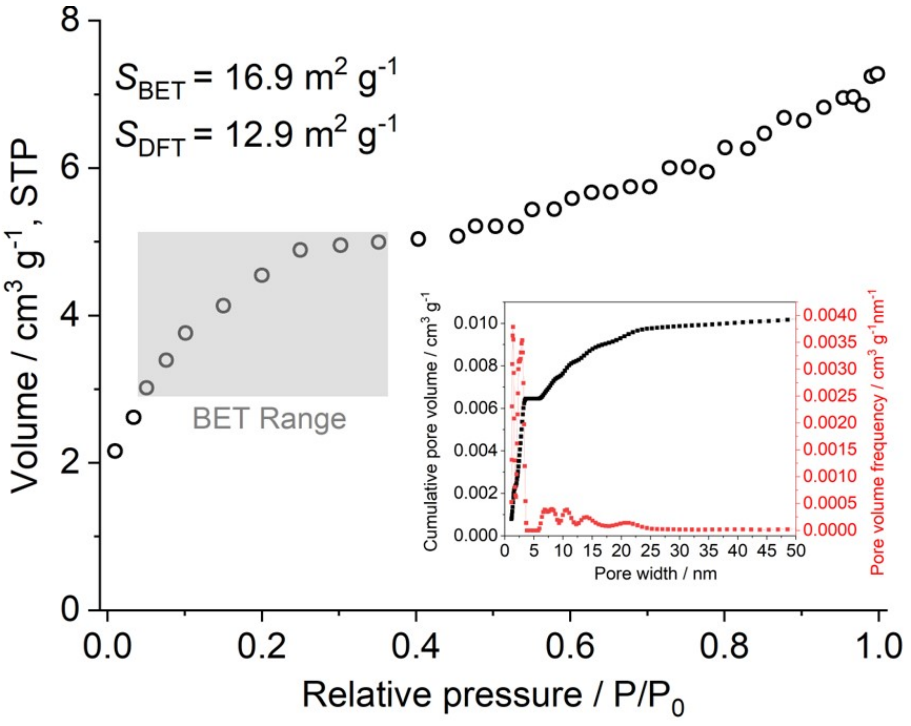


**Figure S7**. $N_2$ gas physisorption measurements with isotherm, pore size distribution, and cumulative pore volume. The surface area is calculated using both BET and (NL)DFT models.

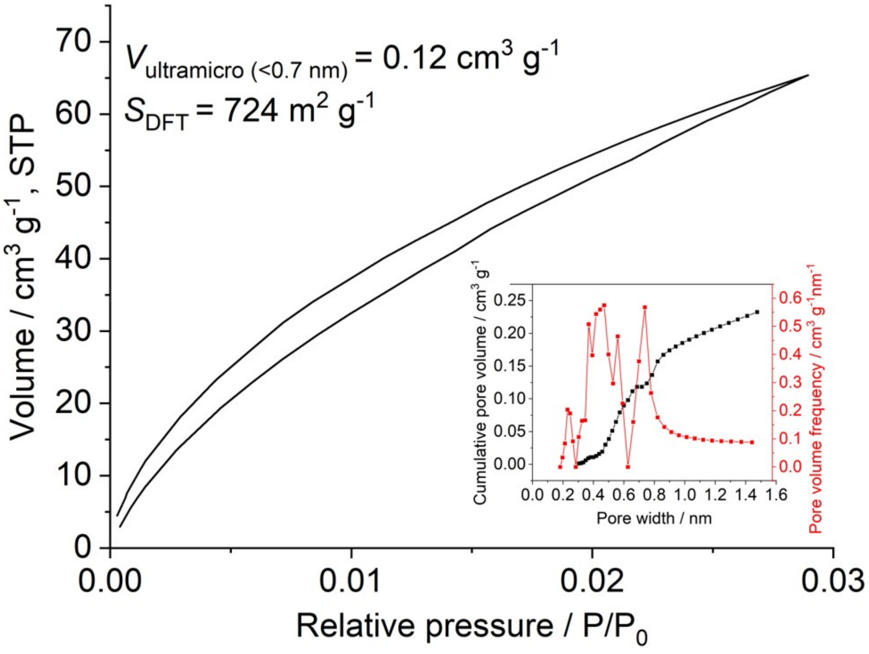


**Figure S8**. $CO_2$ gas physisorption measurements with isotherm, pore size distribution, and cumulative pore volume. The surface area is calculated using the (NL)DFT model.

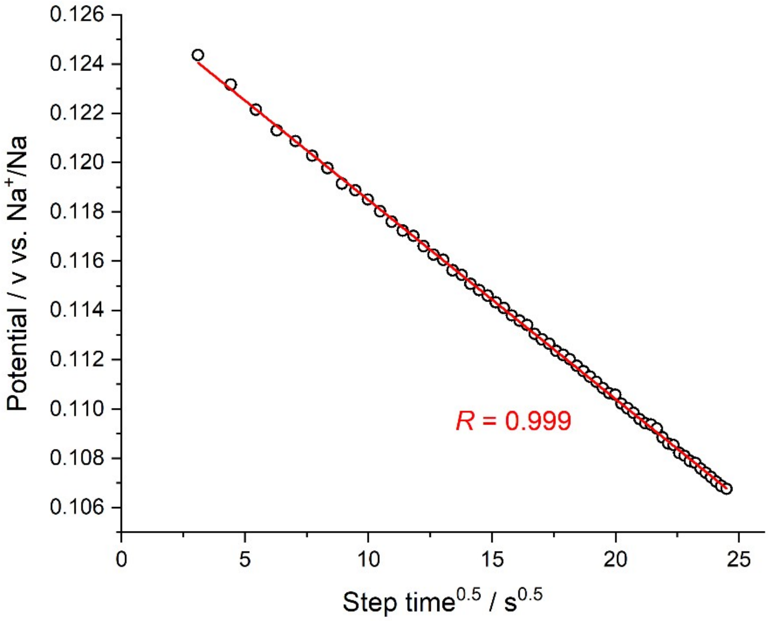


**Figure S9**. Linear fit of the single-step GITT pulse to simplify the first-order approximation.

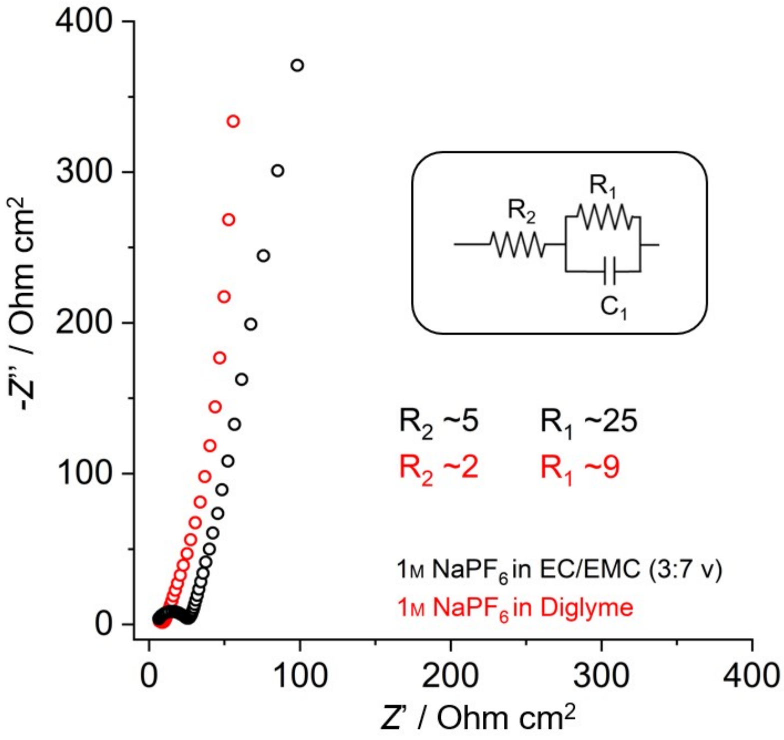


**Figure S10**. Nyquist plots from EIS of HMF-HC under ether- and carbonate-based electrolytes show significantly lower charge transfer resistance in the ether-based electrolyte. Inset: Associated Randle's circuit model and fitting results.

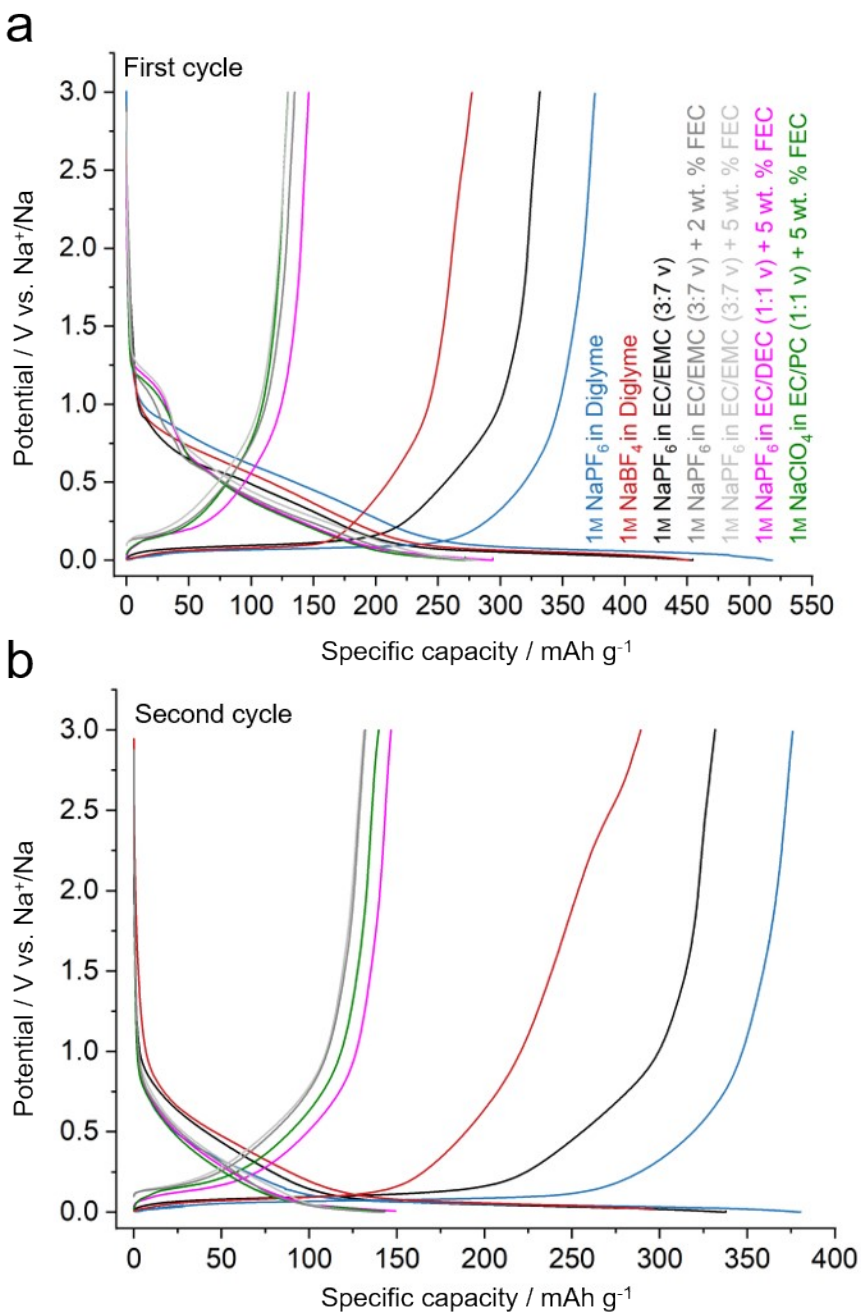


**Figure S11**. GCD measurements of the HMF-HC under different electrolyte environments. (a) Initial cycle. (b) Second cycle. (Current density, 30 mA $g^{-1}$)

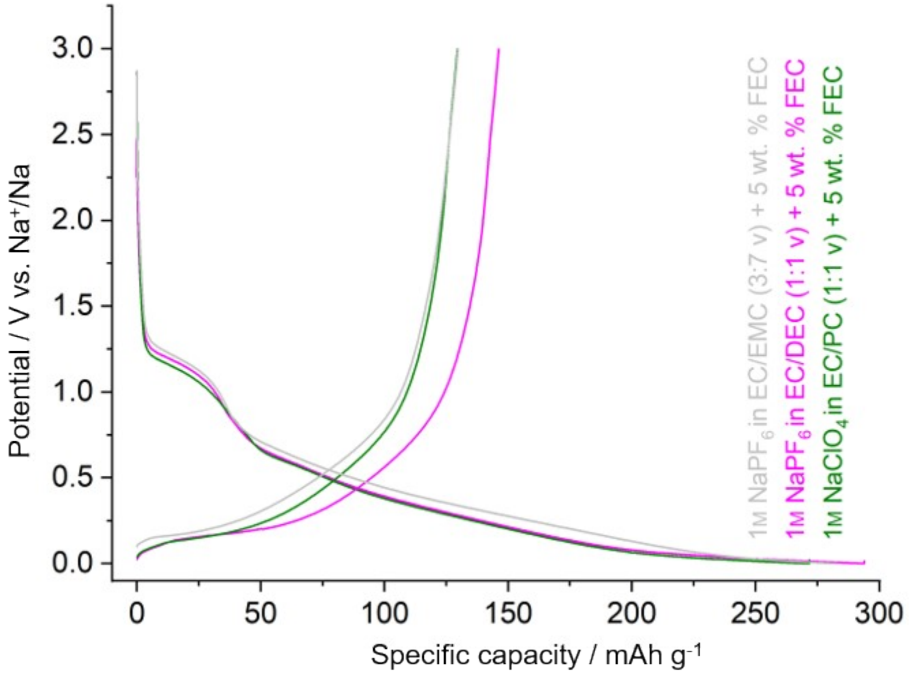


**Figure S12**. GCD measurements of the HMF-HC under different electrolytes with the same amount of FEC additive. (Current density, 30 mA $g^{-1}$)

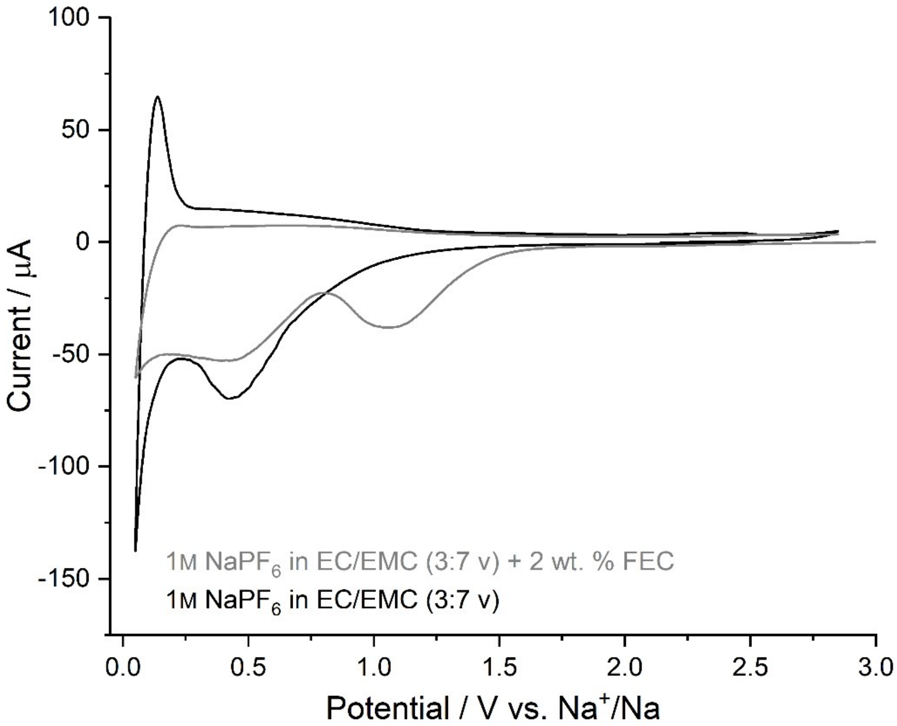


**Figure S13**. Comparison of initial cycle CV at 0.025 mV $s^{-1}$ for HMF electrodes in the in-plane cell, with and without FEC.

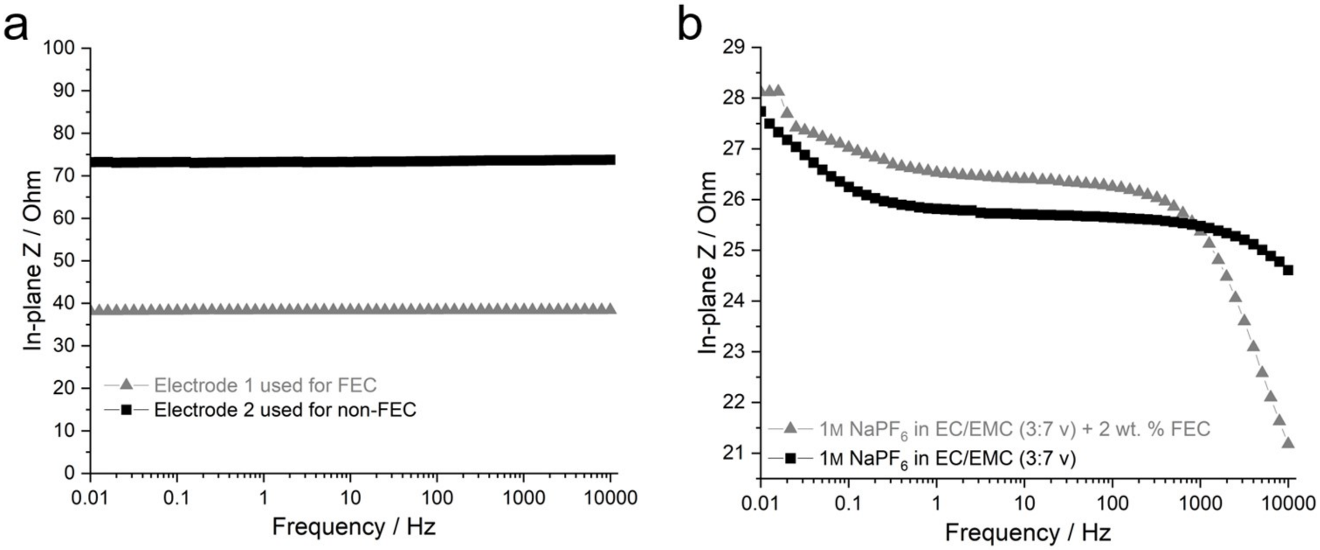


**Figure S14**. (a) Impedance variation with the frequency of dry HMF electrodes. (b) Impedance variation with the frequency of the same electrodes at open circuit voltage under an electrolyte environment.

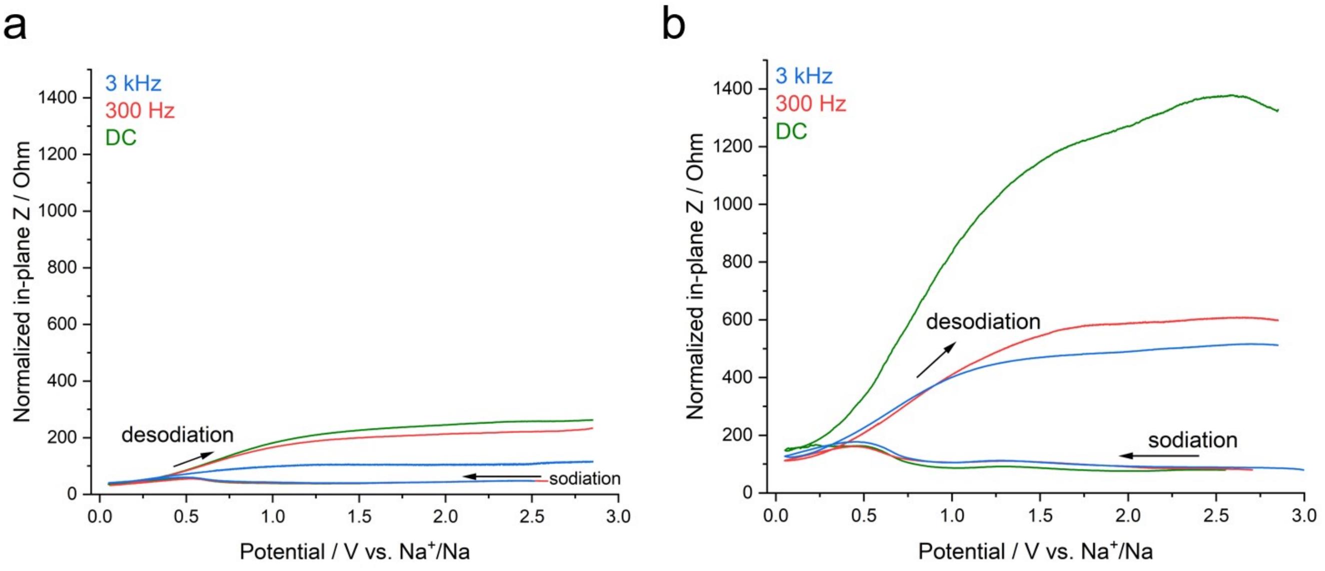


**Figure S15**. Impedance/resistance measurements of HMF electrode in 1 M $NaPF_6$ in EC/EMC at different frequencies: (a) without FEC additive and (b) with FEC additive.

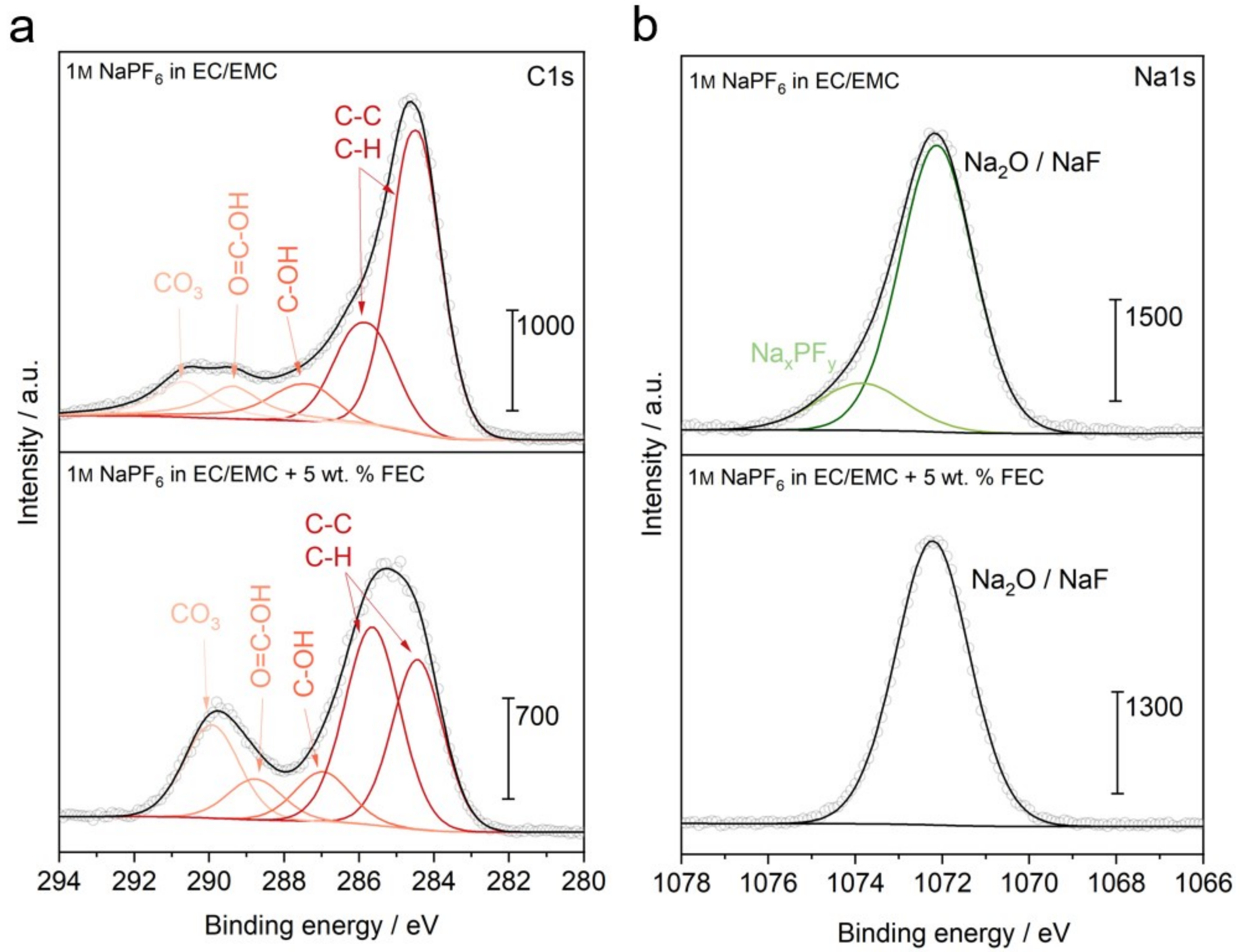


**Figure S16**. *Ex-situ* XPS analysis of the HMF-HC electrode cycled with different electrolytes. (a) C1s and (b) Na1s core-levels.

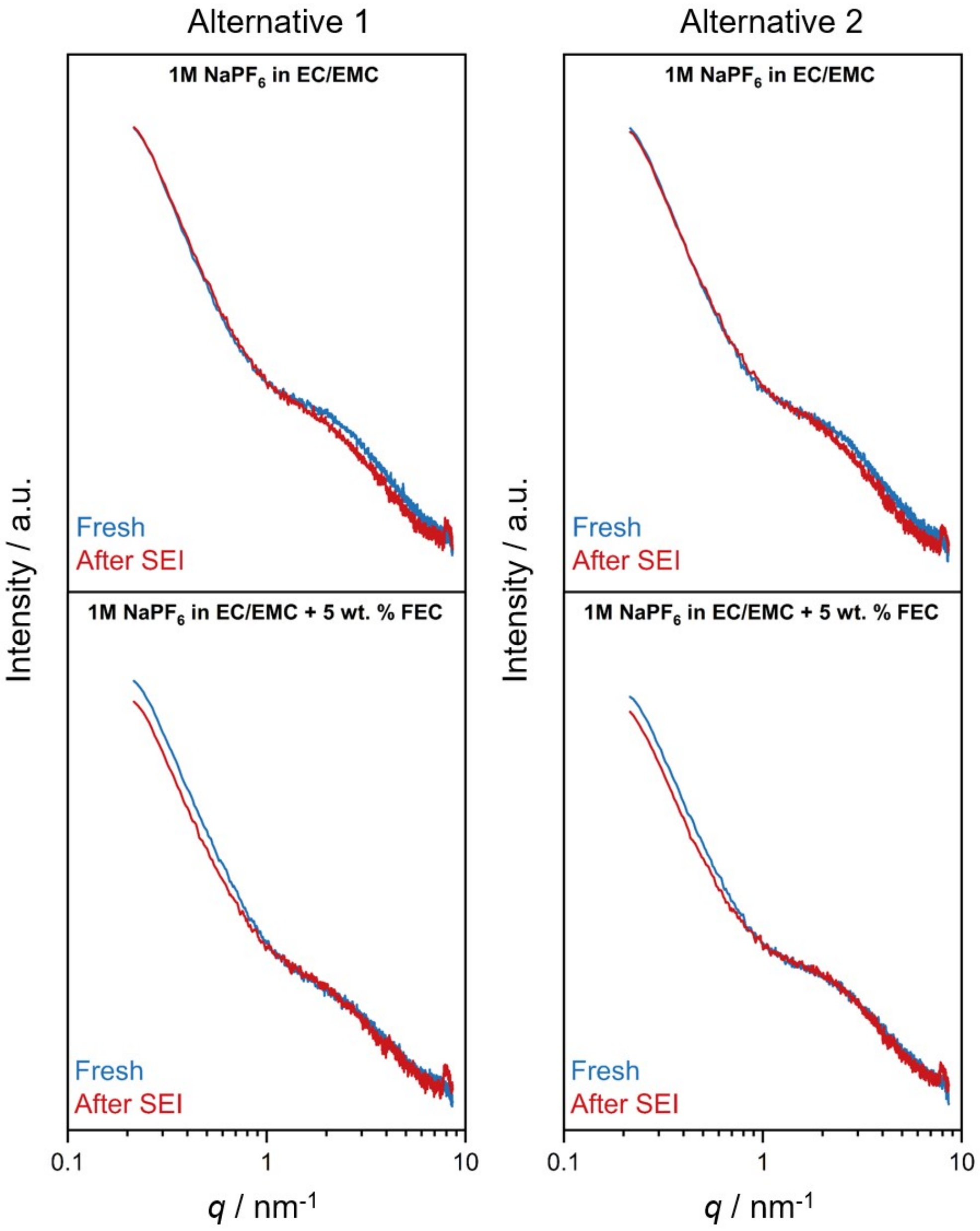


**Figure S17**. Reproducibility measurements regarding the *ex-situ* SAXS of SEI in the presence of FEC additive.

# Supplementary Tables

**Table S1.** Comparison of the main performance metrics and synthesis approaches for the highest-capacity hard carbons reported in SIBs to date.

| Material | Synthesis | Temp. | Maximum stable capacity / mAh $g^{-1}$ | Plateau capacity / mAh $g^{-1}$ | Capacity at high current densities | Full-cell | Ref. |
|---|---|---|---|---|---|---|---|
| **HMF** | One-step heat treatment | 1100°C | ~380 (0.1C) | ~280 (0.1C) | ~300 (1C)<br>~250 (2C) | with NVP | This work |
| **Shaddock peel** | One-step heat treatment | 1200°C | ~400 (0.1C) | ~220 (0.1C) | ~150 (1C)<br>~100 (2C) | n/a | [5] |
| **Esterified starch** | Two-step heat treatment | 300°C (pre.)<br>1100°C | ~370 (0.1C) | ~210 (0.1C) | ~200 (1C)<br>~100 (2C) | with NVP | [6] |
| **Charcoal derived from wood** | Two-step heat treatment | 800°C (pre.)<br>1900°C | ~400 (0.05C) | ~330 (0.05C) | ~190 (1C)<br>~120 (2C) | with NCFM | [7] |
| **Phenol-formaldehyde resin + EtOH (pore-forming agent)** | Two-step heat treatment | 180°C (pre.)<br>1550°C | ~410 (0.1C) | ~280 (0.1C) | ~70 (1C)<br>~50 (2C) | with NaNFM | [8] |
| **Glucose + MgO (template)** | Two-step heat treatment | 600°C (pre.)<br>1500°C | ~450 (0.1C) | ~400 (0.1C) | ~400 (1C) | n/a | [9] |
| **Phenolic resin + PVA (pore-forming agent)** | Two-step heat treatment | 800°C (pre.)<br>1500°C | ~380 (<0.05C) | ~280 (<0.05C) | ~350 (1C) | n/a | [10] |
| **Cellulose** | Two-step heat treatment | 275°C (pre.)<br>1500°C | ~350 (<0.1C) | ~280 (<0.1C) | n/a | n/a | [11] |
| **Epoxy phenol novolac resin + maleic anhydride** | Three-step heat treatment | 180°C (pre. 1)<br>500°C (pre. 2)<br>1800°C | ~480 (0.1C) | ~360 (0.1C) | ~420 (1C)<br>~380 (2C) | n/a | [12] |
| **Activated carbon + Mesoporous carbon** | Two-step heat treatment | 700°C (CVD)<br>1300°C | ~410 (0.1C) | ~300 at 0.1C | ~340 (1C)<br>~290 (2C) | n/a | [13] |

**Table S2**: Elemental compositions of the HMF-HC in wt. %.

| | **C** | **H** | **O** |
|---|---|---|---|
| **Combustive EA** | 97.0 | <0.1 | 2.9 |
| **XPS** | 96.1 | n/a | 3.9 |
| **SEM-EDX** | 97.5 ± 1.0 | n/a | 2.5 ± 1.0 |

**Table S3**: Performance summary of the HMF-HC at different current densities.

| | Current density / mA $g^{-1}$ | | | | |
|---|---|---|---|---|---|
| | **30** | **60** | **150** | **300** | **600** |
| **Desodiation capacity / mAh $g^{-1}$** | 376 | 351 | 328 | 299 | 252 |
| **Desodiation capacity below 0.2 V (vs. $Na^+$/Na) / mAh $g^{-1}$** | 279 | 252 | 235 | 212 | 170 |
| **Average Coulombic efficiency / %** | 98.6 | 99.1 | 99.4 | 99.6 | 99.5 |

**Table S4**: Elemental compositions of the hard carbons from SEM-EDX in wt. %.

| | **C** | **H** | **O** |
|---|---|---|---|
| **HMF-HC** | 97.5 ± 1.0 | n/a | 2.5 ± 1.0 |
| **Glucose-HC** | 95.2 ± 0.7 | n/a | 4.8 ± 1.1 |
| **Fructose-HC** | 96.0 ± 1.5 | n/a | 4.0 ± 0.3 |

**Table S5**: Performance of the HMF-HC//NVP full-cell.

| | **Battery capacity / mAh** | **Average discharge voltage / V** | **Energy density / Wh kg$^{-1}$** |
|---|---|---|---|
| **0.2C** | 0.27 | 3.05 | 217 |
| **0.5C** | 0.23 | 2.96 | 176 |
| **1.0C** | 0.19 | 2.90 | 146 |

**Table S6**: Initial and second cycle performances of HMF-HC in different electrolyte environments based on its initial Coulombic efficiency (ICE, %), total desodiation capacity (mAh $g^{-1}$), and desodiation capacity (mAh $g^{-1}$) below 0.2V (vs. $Na^+$/Na). (Current density, 30 mA $g^{-1}$)

| | **Initial cycle** | | **Second cycle** | |
|---|---|---|---|---|
| | **Desodiation capacity** | **ICE** | **Desodiation capacity** | **Desodiation capacity (<0.2V)** |
| **1M $NaPF_6$ in Diglyme** | 376 | 72 | 375 | 279 |
| **1M $NaBF_4$ in Diglyme** | 277 | 61 | 288 | 158 |
| **1M $NaPF_6$ in EC/EMC (3:7 v)** | 331 | 72 | 331 | 213 |
| **1M $NaPF_6$ in EC/EMC (3:7 v) + 2 wt. % FEC** | 134 | 47 | 131 | 38 |
| **1M $NaPF_6$ in EC/EMC (3:7 v) + 5 wt. % FEC** | 129 | 45 | 130 | 35 |
| **1M $NaPF_6$ in EC/DEC (1:1 v) + 5 wt. % FEC** | 146 | 50 | 146 | 60 |
| **1M $NaClO_4$ in EC/PC (1:1 v) + 5 wt. % FEC** | 129 | 48 | 140 | 46 |

**Table S7**: Resistivity values (in Ohm cm$^2$) of electrodes before and after SEI formation showing the trend in three different electrolytes. The associated Randles circuit model can be found in **Figures 4d** and **4e** of the main text.

| | **Before SEI** | | **After SEI** | | |
|---|---|---|---|---|---|
| | $R_1$ | $R_2$ | $R_1$ | $R_2$ | $R_{SEI}$ |
| **1M $NaPF_6$ in EC/EMC (3:7 v)** | 25.2 | 5.0 | 336.9 | 5.6 | 190.5 |
| **1M $NaPF_6$ in EC/EMC (3:7 v) + 2 wt. % FEC** | 109.8 | 5.5 | 576.3 | 6.6 | 254.5 |
| **1M $NaPF_6$ in EC/EMC (3:7 v) + 5 wt. % FEC** | 116.3 | 5.3 | 1657.0 | 5.7 | 416.3 |

**Table S8**: Elemental compositions (in at. %) of the SEI layer from *ex-situ* XPS.

| | **C** | **O** | **F** | **P** | **Na** |
|---|---|---|---|---|---|
| **1M $NaPF_6$ in EC/EMC (3:7 v)** | 63.8<br>(3.7 as $-CO_3$) | 27.1 | 2.6<br>(n/a as NaF) | <0.5 | 6.0 |
| **1M $NaPF_6$ in EC/EMC (3:7 v) + 5 wt. % FEC** | 49.8<br>(8.8 as $-CO_3$) | 30.0 | 10.0<br>(3.2 as NaF) | <0.5 | 9.7 |

## Supplementary References


[1] W. Weppner, R. A. Huggins, *J. Electrochem. Soc.* **1977**, *124* (10), 1569.

[2] C. Delacourt, M. Ati, J. M. Tarascon, *J. Electrochem. Soc.* **2011**, *158* (6), A741.

[3] a) J. S. Horner, G. Whang, D. S. Ashby, I. V. Kolesnichenko, T. N. Lambert, B. S. Dunn, A. A. Talin, S. A. Roberts, *ACS Appl. Energy Mater.* **2021**, *4* (10), 11460-11469; b) Z. Jian, Z. Xing, C. Bommier, Z. Li, X. Ji, *Adv. Energy Mater.* **2016**, *6* (3), 1501874; c) K. Wang, Y. Jin, S. Sun, Y. Huang, J. Peng, J. Luo, Q. Zhang, Y. Qiu, C. Fang, J. Han, *ACS Omega* **2017**, *2* (4), 1687-1695; d) Y. Li, Y.-S. Hu, M.-M. Titirici, L. Chen, X. Huang, *Adv. Energy Mater.* **2016**, *6* (18), 1600659.

[4] E. Deiss, *Electrochim. Acta* **2005**, *50* (14), 2927-2932.

[5] N. Sun, H. Liu, B. Xu, *J. Mater. Chem. A* **2015**, *3* (41), 20560-20566.

[6] M. Song, Z. Yi, R. Xu, J. Chen, J. Cheng, Z. Wang, Q. Liu, Q. Guo, L. Xie, C. Chen, *Energy Storage Mater.* **2022**, *51*, 620-629.

[7] C. Zhao, Q. Wang, Y. Lu, B. Li, L. Chen, Y.-S. Hu, *Science Bulletin* **2018**, *63* (17), 1125-1129.

[8] Q. Meng, Y. Lu, F. Ding, Q. Zhang, L. Chen, Y.-S. Hu, *ACS Energy Lett.* **2019**, *4* (11), 2608-2612.

[9] A. Kamiyama, K. Kubota, D. Igarashi, Y. Youn, Y. Tateyama, H. Ando, K. Gotoh, S. Komaba, *Angew. Chem. Int. Ed.* **2021**, *60* (10), 5114-5120.

[10] A. Kamiyama, K. Kubota, T. Nakano, S. Fujimura, S. Shiraishi, H. Tsukada, S. Komaba, *ACS Appl. Energy Mater.* **2020**, *3* (1), 135-140.

[11] H. Yamamoto, S. Muratsubaki, K. Kubota, M. Fukunishi, H. Watanabe, J. Kim, S. Komaba, *J. Mater. Chem. A* **2018**, *6* (35), 16844-16848.

[12] C. Fan, R. Zhang, X. Luo, Z. Hu, W. Zhou, W. Zhang, J. Liu, J. Liu, *Carbon* **2023**, *205*, 353-364.

[13] X. Chen, N. Sawut, K. Chen, H. Li, J. Zhang, Z. Wang, M. Yang, G. Tang, X. Ai, H. Yang, Y. Fang, Y. Cao, *Energy Environ. Sci.* **2023**, *16* (9), 4041-4053.
